# Multimodal neurobehavioral integration in binocular color rivalry: cortical-eye movement analysis under color, location, and combined stimuli

**DOI:** 10.3389/fnins.2025.1664583

**Published:** 2025-10-21

**Authors:** Xinni Zhang, Zhineng Lv, Xuesong Jin, Lijun Yun, Guihong Zhao, Zaiqing Chen

**Affiliations:** ^1^School of Information Science and Technology, Yunnan Normal University, Kunming, China; ^2^Department of Education of Yunnan Province, Engineering Research Center of Computer Vision and Intelligent Control Technology, Kunming, China; ^3^Yunnan Key Laboratory of Optoelectronic Information Technology, Kunming, China; ^4^Yuxi Key Laboratory of Mental Health Examination, Yuxi Second People’s Hospital, Yuxi, Yunnan, China

**Keywords:** eye tracking, fNIRS, reaction time, PFC, coupling relationship

## Abstract

**Objective:**

Color and spatial location are key cues influencing visual selection in binocular color rivalry, jointly modulating attentional resource allocation and prefrontal cortex (PFC) activity. However, how these cues—individually and in combination—regulate ocular behavior and PFC activation remains insufficiently understood and lacks systematic empirical investigation.

**Methods:**

This study integrates eye tracking, functional near-infrared spectroscopy (fNIRS), and reaction time measurements to systematically investigate the cortical and oculomotor response characteristics under a binocular rivalry paradigm, focusing on color rivalry (Color stimuli), spatial location (Location stimuli), and their combined (Color & Location stimuli).

**Results:**

The results revealed that Color stimuli elicited rapid saccades, significant pupil dilation, and a decrease in prefrontal HbO concentration. Location stimuli induced stable saccadic patterns and a typical biphasic HbR response in BA46. The combined Color & Location stimuli triggered significant changes in oculomotor behavior during the later processing stage, accompanied by a marked increase in HbO activation in BA10, suggesting its dominant role in multisensory integration and cognitive resource reallocation. Further coupling analyses showed a significant positive correlation between prefrontal HbO concentration and reaction time (*r* = 0.555, *p* < 0.01), and a significant negative correlation between HbO concentration and saccade amplitude (*r* = –0.376, *p* < 0.05), consistent with the theoretical predictions of the “neural efficiency–cognitive load trade-off” model. Task-dependent coupling relationship were also observed among oculomotor parameters and between eye movement and cerebral hemodynamic signals.

**Conclusion:**

Color stimuli induce rapid saccadic behavior and impose higher prefrontal load, Location stimuli engage a more efficient dorsal pathway, while Color & Location stimuli intensify resource rivalry and induce a processing bottleneck, manifested as prolonged reaction times co-occurring with heightened cortical activation.

## 1 Introduction

In the human stereoscopic visual system, when the corresponding retinal positions of the two eyes receive different color stimuli, if the color difference is below the fusion threshold of the visual system, the observer perceives a stable and fused color experience. This phenomenon is known as binocular color fusion. Conversely, when the color difference exceeds this threshold, the inputs from the two eyes enter perceptual rivalry, resulting in spontaneous alternations between the two-color percepts — a phenomenon referred to as binocular color rivalry (Jung et al., 2011; Malkoc and Kingdom, 2012). This perceptual phenomenon not only reflects the fundamental inhibitory mechanisms in the visual cortex but has also become a critical paradigm for understanding conscious perception switching. Research on binocular color rivalry has evolved across multiple levels—from psychophysical laws to neural mechanisms and computational modeling.

Early studies on binocular rivalry primarily focused on the psychophysical relationship between stimulus attributes and perceptual outcomes, such as mutual inhibition between color channels, interactions between luminance and neural antagonism, and the rules revealed by Levelt’s laws regarding the relationship between stimulus strength and dominance duration (Breese, 1899; Gellhorn and Schöppe, 1925; Levelt, 1965). Subsequent research further demonstrated that the rivalry process not only occurs in the primary visual cortex (V1) but is also subject to feedback regulation from higher-order brain regions such as the prefrontal cortex (PFC). Moreover, color plays a specific role in rivalry: it can enhance visual integration capacity, modulate the rate of perceptual alternation through rhythmic PFC activity, and enable flexible switching between fusion and rivalry states under task demands (Blake and Fox, 1974; Li et al., 2013; Brascamp et al., 2015; Riesen et al., 2019; Chen et al., 2019). More recently, modeling and machine learning approaches have revealed the coupling effects of visual cues such as color and disparity, highlighting the critical role of the occipito-parietal network in discriminating between fusion and rivalry states (Lv et al., 2024).

Spatial location information refers to changes in the position of a stimulus along the horizontal or vertical axis, for example, when the same stimulus moves from the left to the right side of the screen in a visual task. As an important component of visual attention, it also plays a critical modulatory role in perceptual integration. Existing studies indicate that location changes not only reconfigure saccade paths but may also increase spatial working memory load, thereby activating the dorsolateral prefrontal cortex (DLPFC) (Courtney et al., 1997; Owen et al., 2005). In contrast, color changes are more likely to trigger semantic processing and attentional control, engaging the ventrolateral prefrontal cortex (VLPFC) (Corbetta and Shulman, 2002). When both color and location cues change simultaneously, the visual system must coordinate multiple sources of perceptual conflict, mobilizing additional cognitive resources to achieve effective suppression and integration (Botvinick et al., 2001; Desimone and Duncan, 1995). Although Stromeyer et al. (1992) revealed the coupling mechanism between color fusion thresholds and spatial sensitivity through psychophysical experiments and established a quantitative model of interaction between chromatic and spatial information, their conclusions were primarily based on static conditions. Systematic investigations of the interaction between dynamic location changes and color in the context of dynamic binocular rivalry remain insufficient. Recently, Zhang et al. (2025) used eye-tracking to show that saccadic characteristics in dynamic binocular rivalry differ significantly from those in static fusion, suggesting that the temporal dynamics of color and location coupling influence perception and attention. However, comprehensive evidence regarding their combined effects on perceptual alternation rate, attentional allocation, and neural responses remains limited.

To address this research gap, the present study employs a simultaneous recording approach combining functional near-infrared spectroscopy (fNIRS) and high-precision eye tracking. fNIRS enables monitoring of hemodynamic changes in brain regions such as the PFC with relatively high temporal resolution, offering strong ecological validity and robust resistance to motion artifacts. Eye tracking allows precise measurement of behavioral indices such as fixation position, saccade trajectories, and pupillary responses. The combined use of these two techniques has been widely applied in visual cognitive research and has proven effective in simultaneously revealing neural activity and behavioral characteristics related to visual attention, enabling quantitative analysis of attentional allocation and decision-making processes (Wang et al., 2025; Yu et al., 2025; Xu et al., 2025). In binocular rivalry tasks, the transition from fusion to rivalry is often accompanied by eye movements such as gaze shifts and micro saccades. Color and location changes may preferentially trigger spatial updating or feature re-binding at different stages of these events, yet systematic evidence for such dynamic interactions at both neural and behavioral levels remains lacking.

Based on the above theoretical and empirical background, this study proposes three hypotheses:

(1)    Stimuli involving color changes will elicit saccadic responses and increased prefrontal cognitive load, particularly reflected in significant activation of the VLPFC;
(2)    Stimuli involving location changes will preferentially activate the dorsal pathway to support efficient spatial information updating and maintenance;
(3)    When both color and location cues change simultaneously, the visual system will face intensified resource rivalry and processing bottlenecks, manifested as prolonged reaction times co-occurring with widespread hyperactivation in the PFC.

To test these hypotheses, we designed three stimulus conditions—Color, Location, and Color & Location—and simultaneously recorded fNIRS hemodynamic responses from key prefrontal regions (e.g., BA8, BA9, BA10, BA46) and multiple eye movement parameters (including Fixation Duration, Saccade Amplitude etc.). The study focuses on analyzing the following three aspects:

(1)    Dynamic characteristics of eye movement behavior under the three stimuli conditions;
(2)    Hemodynamic response patterns across PFC regions under varying task loads;
(3)    Coupling relationships among eye movement behavior, fNIRS hemodynamic responses, and reaction time.

We aim to reveal the mechanisms through which multi-source information complexity modulates visual cognitive processing and provide new empirical support for models of attentional control and prefrontal neural coordination.

## 2 Materials and methods

### 2.1 Equipment and experimental environment

**FIGURE 1:**
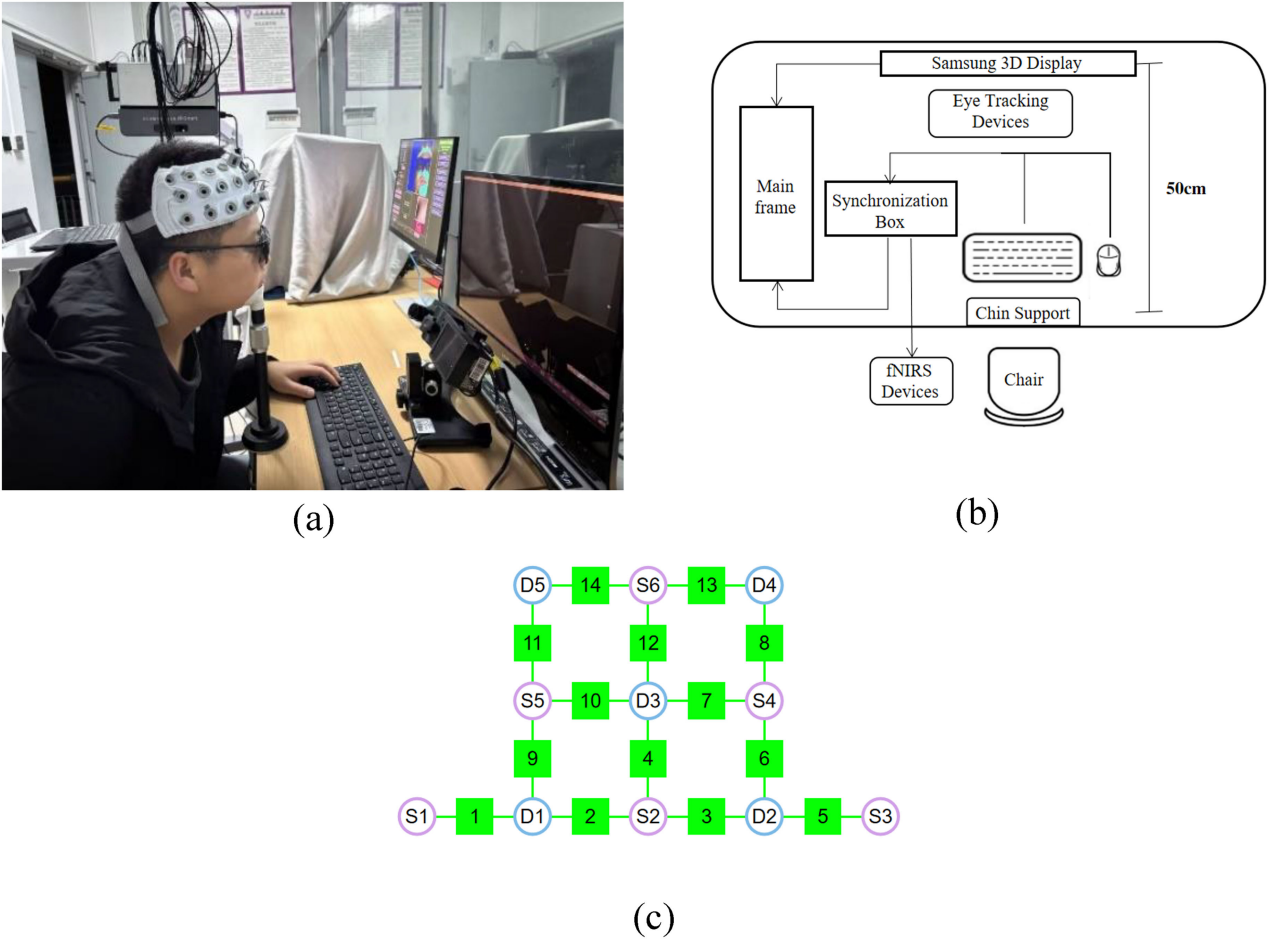
Apparatus and equipment used in the experiments. **(a,b)** Diagram of 0 experimental equipment and apparatus layout **(c)** fNIRS system configuration: S1–S6 denote the sources, D1–D5 represent the detectors, and channels 1–14 are the measurement channels.

As shown in Figure 1a, visual stimuli were presented using the Experiment Builder software on a Samsung 3D display (model: S23A950D) equipped with active shutter 3D glasses. The display has a 2D/3D/3D parallel switching function with a screen area of 511.8 mm (H) × 288.3 mm (V), a resolution of 1920 (horizontal) × 1080 (vertical) pixels, and a refresh rate of 120 Hz. The glasses were active stereoscopic glasses, which could be used to view the 3D stimuli paradigm by pressing a switch button. To produce a stimuli paradigm with specific CIE-1931 coordinates and luminance, display calibration and colorimetric characterization were carried out using the look-up tables (LUT) method. The brightness and chromaticity were measured at the center point of the display through the 3D glasses using a spectroradiometer (Photo Research PR-715), and the characterization accuracy is 1.56 units of CIELAB color difference ($\Delta \,E_{a\,b}^{*}$). In addition, the interocular crosstalk of the display system was quantified (Baker et al., 2016) and the results showed that the crosstalk level was below 3% (defined as the ratio of the light intensity received by the non-target eye to that of the target eye), which meets the accuracy requirements of vision research.

As shown in Figure 1b, A synchronization box was used to control the start and stop of the fNIRS and eye tracking devices, ensuring precise synchronization across all equipment. To minimize interference caused by spontaneous head movements, participants’ heads were stabilized using a chin support. All experiments were conducted in a dark room, and a fixed viewing distance of 50 cm was maintained between the participant and the display throughout the experiment.

Eye movement data were recorded at a sampling rate of 2 kHz using the EyeLink 1000 Plus video-based eye tracker, capturing binocular movement information. Prior to the formal experiment, participants performed a 9-point calibration procedure, with calibration error maintained within 0.5° of visual angle. Following successful calibration, a pre-recording session was conducted to ensure stable and accurate capture of binocular motion data.

fNIRS data were collected using the NirSmart-6000A system (DanYang Huichuang Medical Equipment Co., Ltd., Jiangsu, China). The system consists of a near-infrared light source (light emitting diodes, LED) and an avalanche photodiode (APD) as detectors, with source wavelengths of 730 nm and 850 nm, and a sampling rate of 11 Hz. A total of six light sources and five detectors were used during the experiment, forming 14 effective measurement channels, with a fixed source–detector distance of 3 cm for each channel, as shown in Figure 1c. The probe arrangement followed the international 10–20 electrode system to cover the PFC region of the participant’s brain.

### 2.2 Subjects

Various factors in the visual system, such as visual acuity, can affect the allocation of attention. Therefore, we had to screen the subjects’ visual acuity strictly. Ophthalmologists and specially trained research assistants (author Xinni Zhang) completed the ophthalmic screening of all subjects. Visual acuity was assessed using logarithmic visual acuity scales at a standard distance of 4 meters for both naked eye and corrected visual acuity, and for myopic subjects, a comprehensive optometric instrument was used to accurately quantify their corrected visual acuity. An in-depth visual function test followed the visual acuity assessment. We examined color vision by the subject’s ability to recognize colored disks containing numbers or patterns in the Ishihara Color Blindness Test chart, and the depth and three-dimensional structure of the subject’s vision by looking at pictures consisting of random dots using the Titmus Stereoscopic Chart (stereoscopic sensitivity ≤ 100 arcsec) test. Finally, the presence and exact location of the blind spot were defined by the traditional method, in which a research assistant, holding a small pointer, guided the subject to gaze at a fixed reference in the distance, and then slowly moved the pointer until the subject could no longer track its position.

16 subjects (8 males and 8 females) underwent visual acuity and visual function testing and met the experimental criteria. The subjects were between 22 and 30 years old, 10 of them wore glasses and 6 did not. All subjects had normal visual acuity, normal stereopsis, and no significant refractive errors. Additionally, they were free from color blindness or any form of color vision deficiency, as well as neurological or psychiatric disorders, as confirmed by a standardized health questionnaire and a structured interview.

The sample size (*n* = 16) was determined *a priori* using G*Power 3.1 to ensure sufficient statistical power. A power analysis was conducted for the primary within-subjects effect of interest, assuming a significance level of α = 0.05 and a desired power (1 – β) of 0.80. The analysis indicated that 16 participants would provide adequate power to detect meaningful effects, supporting the robustness of the experimental design.

### 2.3 Stimuli

**FIGURE 2:**
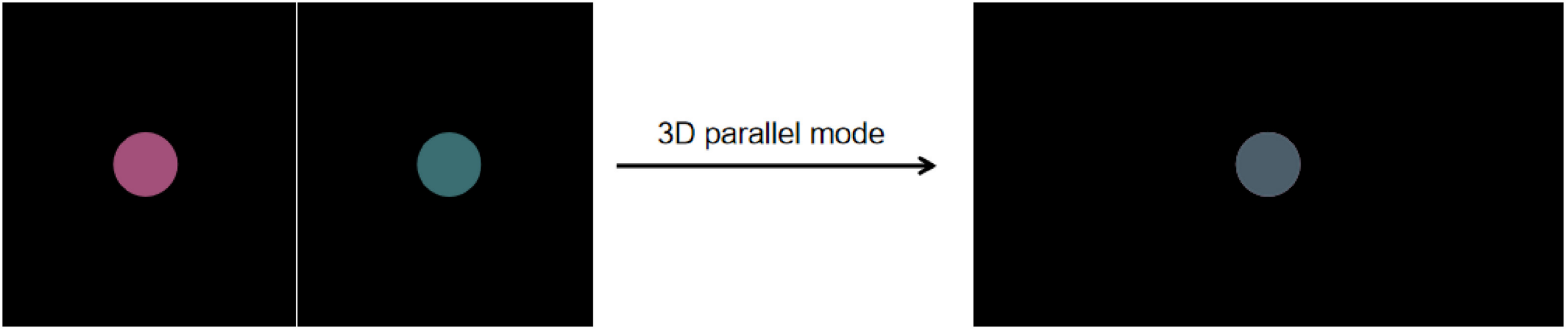
The presentation of the red–green color rivalry pair in the 3D parallel mode. Using the 3D parallel display mode combined with active shutter glasses, images for the left and right eyes were alternately presented, visually fusing into a central disk and thereby inducing binocular color rivalry perception.

This study includes three types of experimental stimuli: Color stimuli, Location stimuli, and Color & Location stimuli. Under the color rivalry condition, different colored disks were presented to the left and right eyes, with both disks fully overlapping in the same retinal region. Using the 3D parallel display mode combined with active shutter glasses, images for the left and right eyes were alternately presented, visually fusing into a central disk and thereby inducing binocular color rivalry perception (Figure 2). Participants could perceive either a single color or a mixed color (alternating red and green). This design ensures that perceptual changes arise from binocular rivalry rather than spatial separation. Under the gray baseline condition, identical gray disks were presented to both eyes to provide a stable baseline. All stimuli in this study were presented using this mode. The design details for these three types of stimuli are described below.

#### 2.3.1 Color stimuli

**FIGURE 3:**
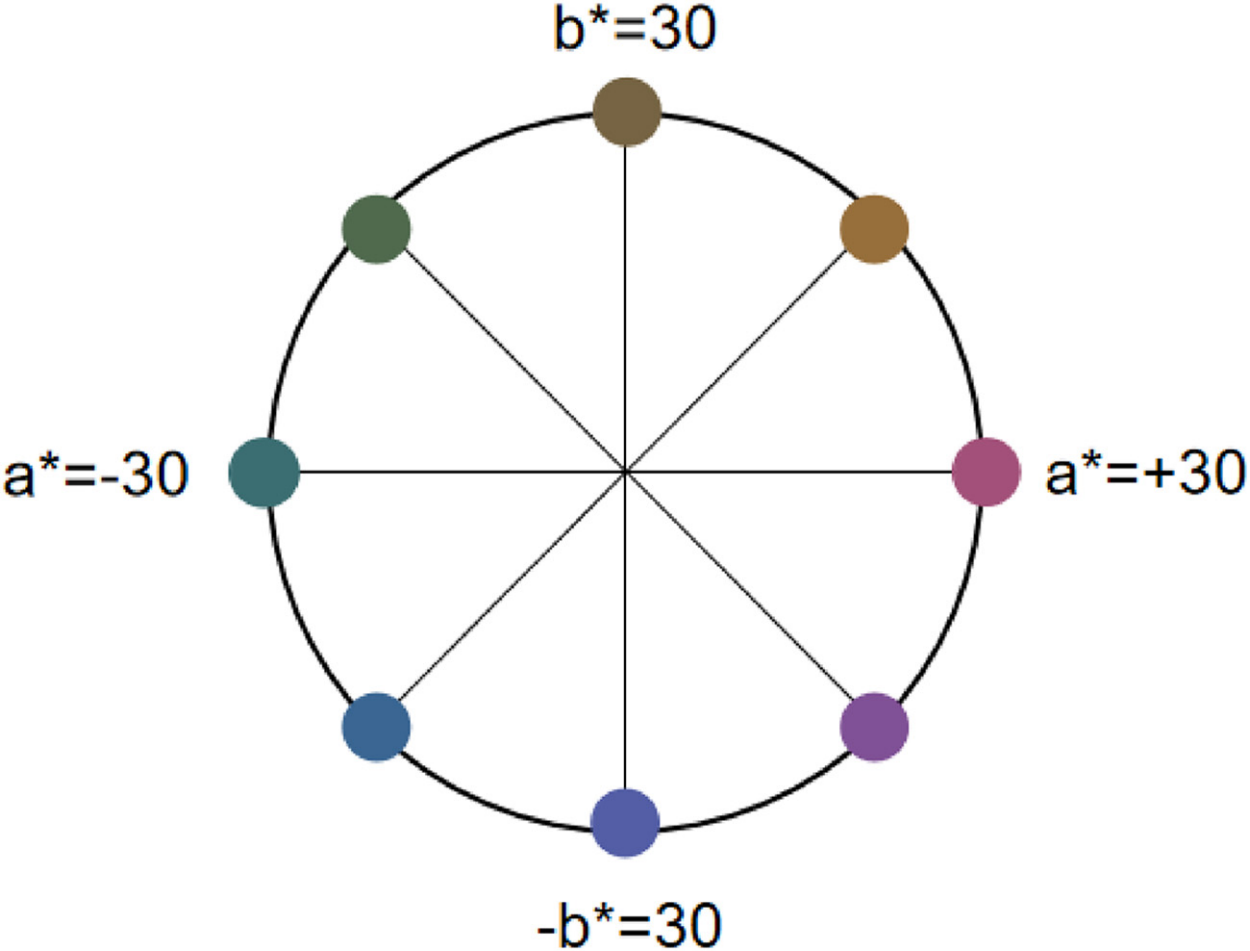
The location of rivalry color pairs in the LAB space. Eight colors from the CIELAB color space with a phase difference of 45° and grouped them into four rivalry color pairs as the Color stimuli. In CIELAB, a* denotes the R-G axis (±30 as offsets toward red/green) and b* the Y-B axis (±30 as offsets toward yellow/blue).

We selected eight colors from the CIELAB color space with a phase difference of 45° and grouped them into four rivalry color pairs as the Color stimuli (Figure 3): Red-Green (R-G), Yellow-Blue (Y-B), Orange-Cyan (RY-GB), and Purple-Yellow Green (RB-GY). All color pairs had an L* value set to 30, and brightness was controlled at 15 ± 0.5 cd/m^2^ (Xiong et al., 2021). Based on the monitor’s color characterization, we measured the XYZ chromaticity values of the selected colors, the coordinates of each color in the CIELAB color space and their corresponding XYZ chromaticity values are listed in Table 1.

**TABLE 1:** Correspondence table of colorimetric lab values and XYZ chromaticity values.

Color	L * a * b * coordinates	X	Y	Z
R	(30, 30, 0)	21.3744	14.9509	19.6127
G	(30, −30, 0)	10.2247	13.3157	17.5568
Y	(30, 0, 30)	12.9745	13.2904	7.1288
B	(30, 0, −30)	14.4834	12.6146	37.7087
RY	(30, 21, 21)	16.45	13.0927	9.3736
GB	(30, −21, −21)	11.7438	12.4282	29.5437
RB	(30, 21, −21)	17.4042	12.6904	30.4557
GY	(30, −21, 21)	9.8424	12.5894	9.0854
GRAY	(46, 0, 0)	50.5868	48.2002	66.6620

**FIGURE 4:**
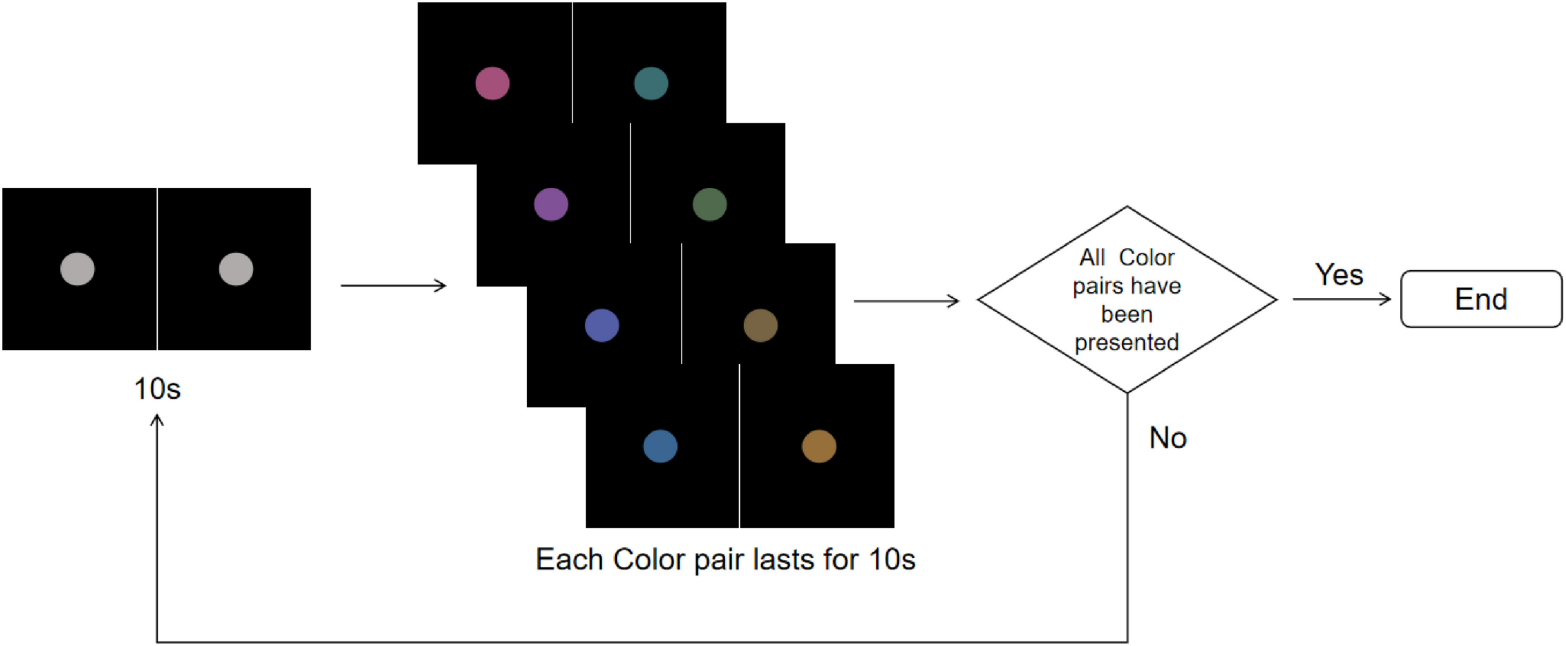
Color stimuli flowchart. The experiment starts with a central gray disk presented for 10 s, followed by the random presentation of one of the four rivalry color pairs (R-G, Y-B, RY-GB, RB-GY) for 10 s, repeated 8 times. The trial ends after all color pairs have been presented. To more clearly illustrate the stimulus design and presentation procedure, the figure shows only the single-eye stimuli in 2D mode. In the actual experiment, the left- and right-eye images were fused into a central disk using the 3D parallel display mode, which was presented to participants.

The stimuli consisted of two disks with a diameter of 2° presented on a black background with a brightness of 0.24 cd/m^2^. When presenting the R-G color pair, one disk displayed red and the other green on either side. Each stimuli lasted 160 s, alternating between gray disks and randomly selected rivalry color pairs. Specifically, this involved 10 s of gray disk followed by 10 s of a color pair, with the sequence of rivalry color pairs being randomized (Figure 4). This process was repeated eight times. To avoid any positional or frequency biases affecting the results, the disks were fixed at the center of the screen throughout the experiment. Participants were instructed to maintain fixation on the stimuli and press the “A” key immediately upon noticing the alternation between the gray field and the color pairs.

#### 2.3.2 Location stimuli

**FIGURE 5:**
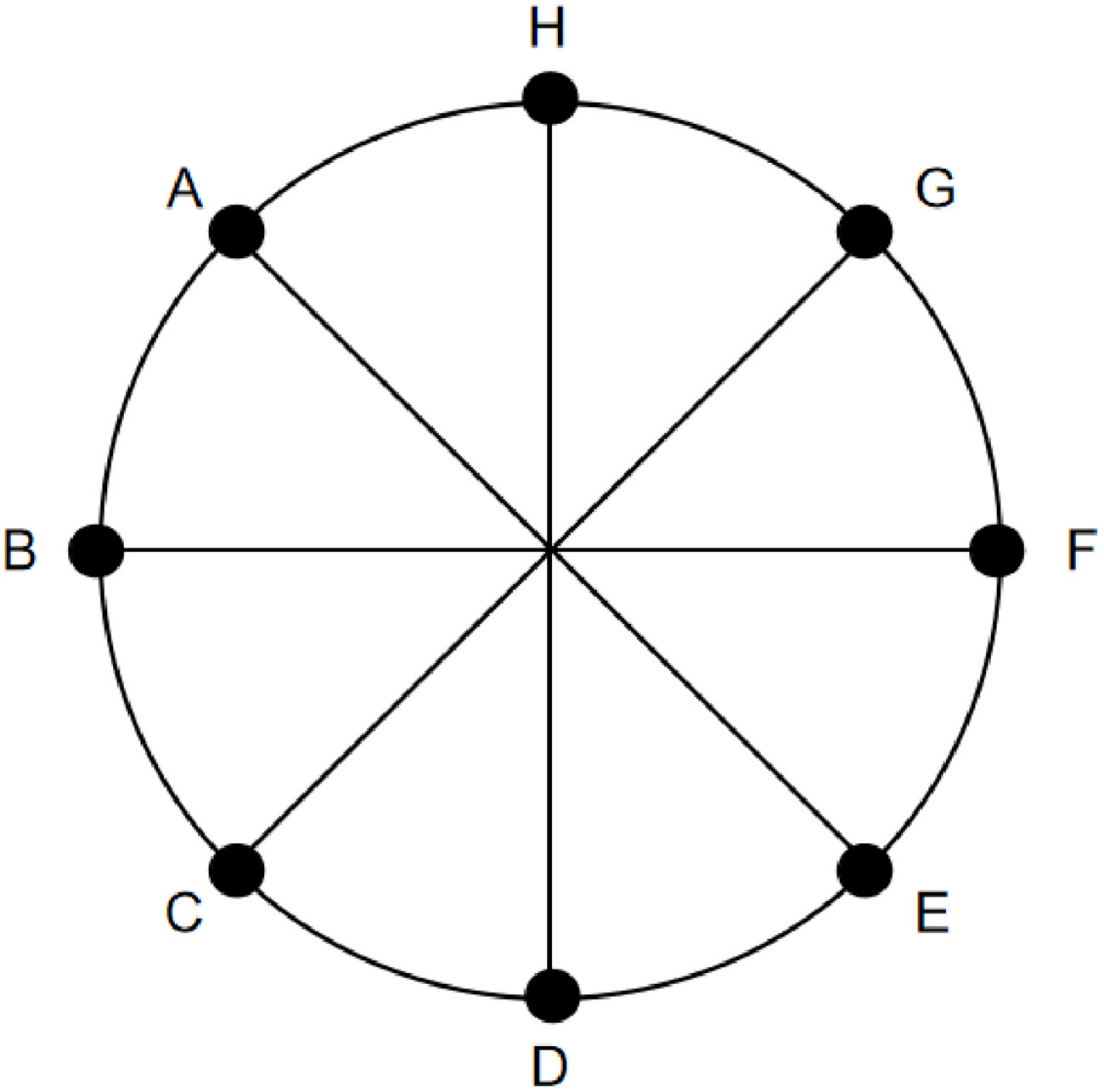
Schematic diagram of Location stimuli selection. Eight equidistant positions along a 14° circular path (labeled **A-H** clockwise, starting from the top as **A**) were selected as the location points for the Location stimuli. During stimulus presentation, these eight positions appeared in a random order.

**FIGURE 6:**
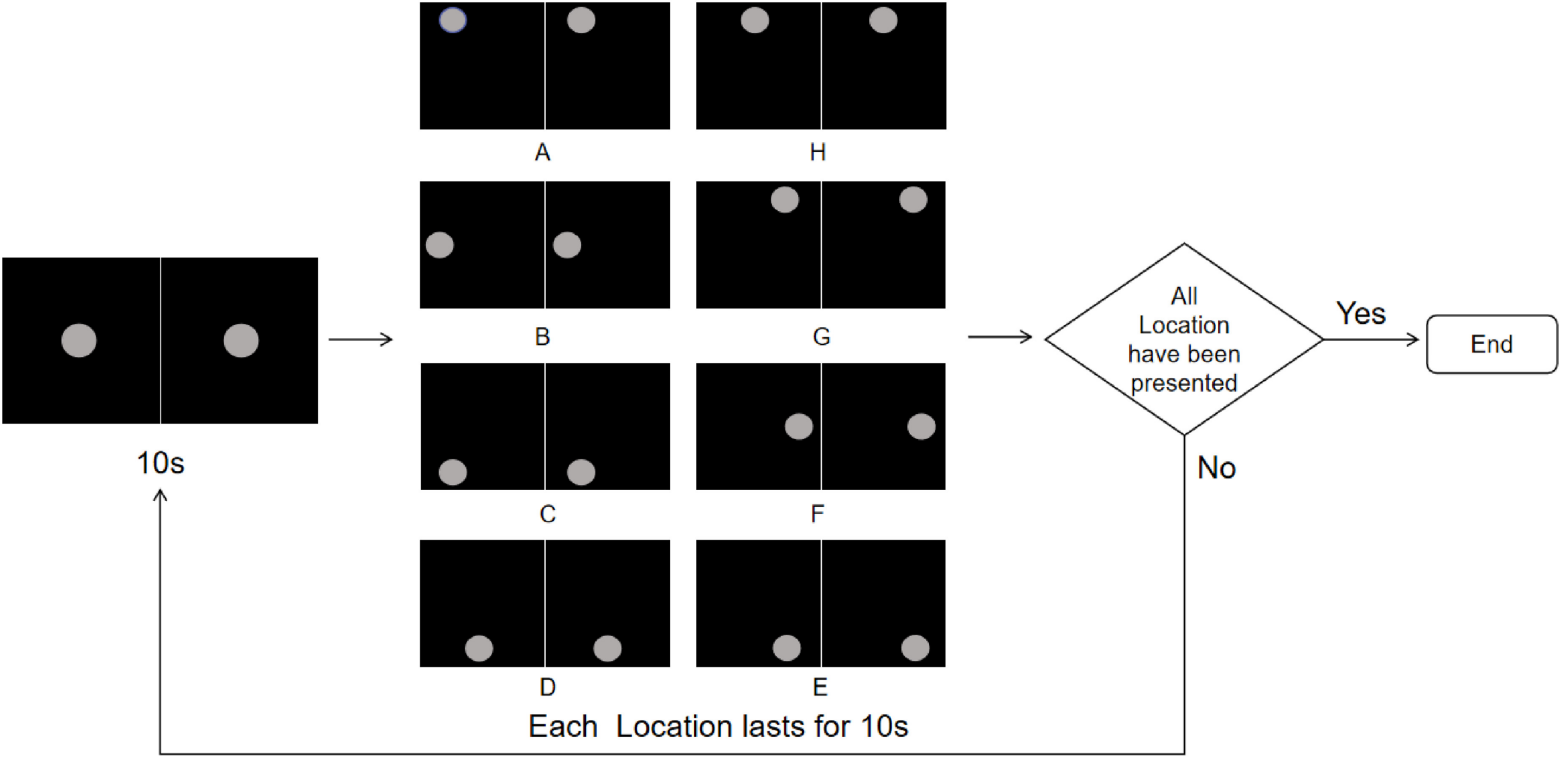
Location stimuli flowchart. The experiment starts with a central gray disk presented for 10 s, followed by random jumps to one of the eight predefined positions **(A-H)** along a 14° circular path, each lasting 10 s, repeated 8 times to ensure each location appears once. The trial ends after all locations have been presented; if not completed, the sequence is repeated. To more clearly illustrate the stimulus design and presentation procedure, the figure shows only the single-eye stimuli in 2D mode. In the actual experiment, the left- and right-eye images were fused into a central disk using the 3D parallel display mode, which was presented to participants.

Eight positions spaced 45° apart along a 14° circular path (designated A-H) were selected as the location points for the Location stimuli (Figure 5). Each stimuli lasted 160 s, starting with a gray disk centered on the screen for 10 s, followed by random jumps to one of the positions A-H for 10 s (Figure 6). This sequence was repeated eight times. To ensure that the number of appearances did not affect the results, each position appeared once. Throughout the experiment, participants were required to maintain fixation on the stimuli and press the “A” key immediately upon observing any change in the disk’s position.

#### 2.3.3 Color & Location stimuli

**FIGURE 7:**
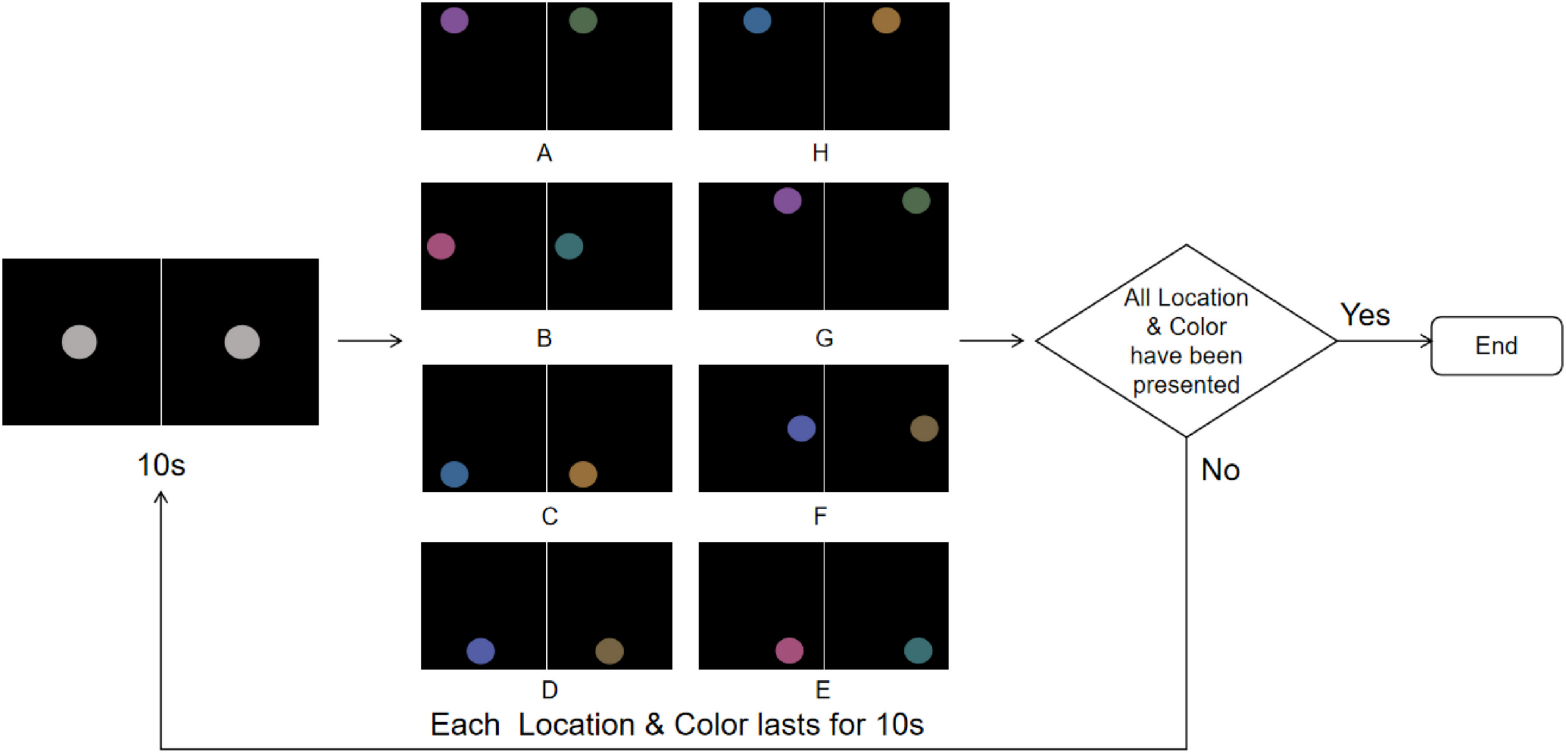
Color & Location stimuli flowchart. The experiment starts with a central gray disk presented for 10 s, followed by random jumps to one of the eight predefined positions **(A-H)** along a 14° circular path while simultaneously changing to one of the four rivalry color pairs (R-G, Y-B, RY-GB, RB-GY) for 10 s, repeated 8 times to ensure equal probability for all Color & Location pairs. The trial ends after all Color & Location combinations have been presented; if not completed, the sequence is repeated. To more clearly illustrate the stimulus design and presentation procedure, the figure shows only the single-eye stimuli in 2D mode. In the actual experiment, the left- and right-eye images were fused into a central disk using the 3D parallel display mode, which was presented to participants.

Under the Color & Location stimuli, both color and location stimuli were presented in a fully randomized manner to combine feature and spatial conflicts while avoiding potential biases due to uneven frequency distribution. Each stimulus session lasted 160 s, beginning with a gray disk presented at the center of the screen for 10 s (Figure 7). Subsequently, the disk would randomly jump to one of the eight pre-defined positions (A–H) along a 14° circular path (with 45° phase intervals) while simultaneously changing to one of the four rivalry color pairs (R-G, Y-B, RY-GB, RB-GY) for 10 s. This random position–color presentation was repeated eight times, ensuring that each position and color pair appeared with equal probability throughout the experiment. Participants were instructed to maintain fixation on the disk and to press the “A” key immediately upon detecting any changes in its position or color, thereby providing synchronous marking of perceptual events.

#### 2.3.4 Procedure

**FIGURE 8:**
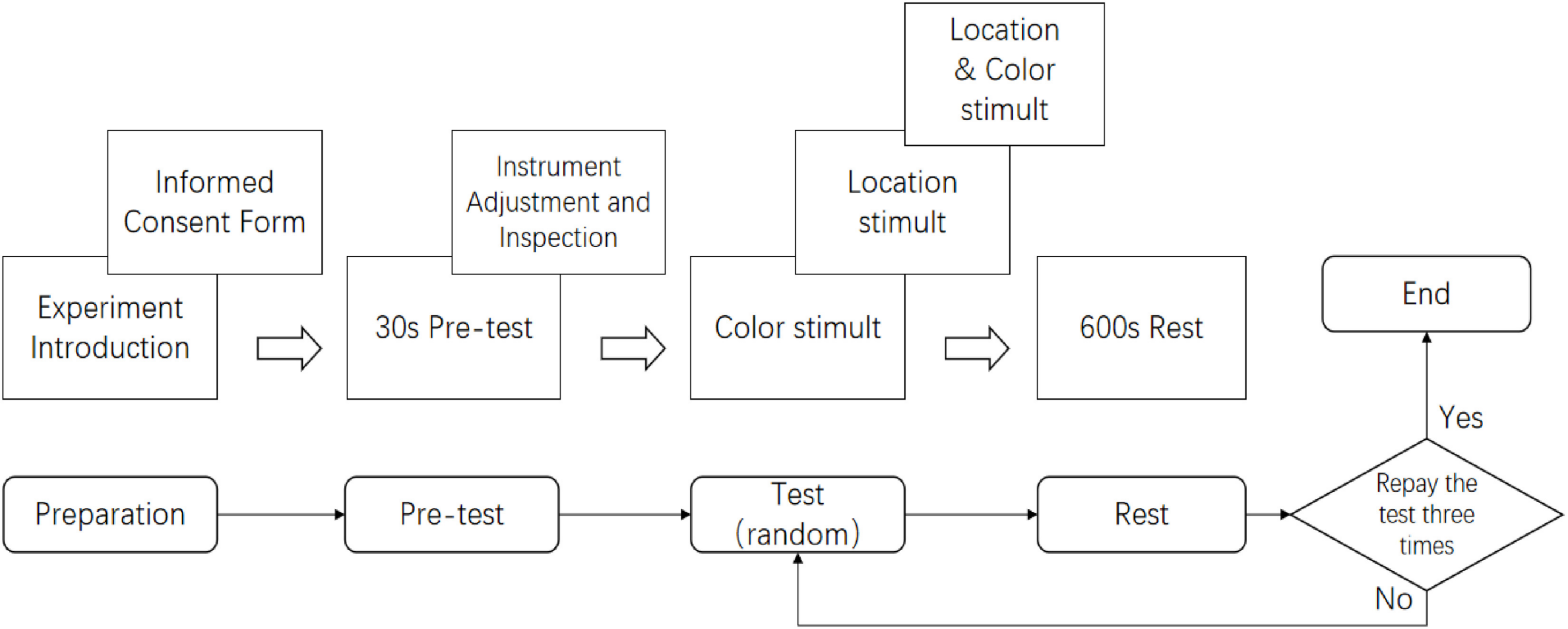
Illustration of the experimental procedure. Participants (one per day at 8 AM) provided informed consent, wore the fNIRS cap and 3D glasses, and completed a 30-s pre-test presenting Color, Location, and Color & Location stimuli in randomized order (170 s each). They pressed key “A” upon detecting binocular color rivalry or location alternation, with responses within 1.5 s required to proceed. Equipment was checked prior to the main experiment.

The experimental procedure is illustrated in Figure 8. To maximize alertness and attention, experiments were conducted daily at 8 AM, with only one participant tested per day. Upon arrival, participants were briefed on the experiment and signed an informed consent form ensuring anonymity (World Medical Association, 2001). Participants then donned the fNIRS cap and 3D glasses and underwent a 30-second pre-test to familiarize themselves with the procedure. During the pre-test, three stimulus conditions (Color, Location, and Color & Location) were presented in randomized order, each lasting 170 s. Participants were instructed to press the response key “A” immediately upon detecting binocular color rivalry or location alternation in the Color or Location conditions, and whenever either a color or location change was detected in the Color & Location condition. Participants had to respond correctly within 1.5 s to proceed to the main experiment. Experimenters ensured that all equipment, including eye-tracking and fNIRS devices, was functioning properly before the main experiment began.

The main experiment consisted of three rounds, each including one set of Color, Location, and Color & Location stimuli. Participants were allowed brief rest periods between rounds. The total experimental duration was approximately 30 min.

### 2.4 Data acquisition and preprocessing

The raw fNIRS data were processed via the analysis software (NirSpark, Huichuang, China). The preprocessing consisted of four steps: (1) eliminating unrelated time intervals from the data; (2) removing the artifacts induced by motion and environment using the automatic motion correction function (standard deviation of the threshold = 6.0 and amplitude of the threshold = 0.5) (Li et al., 2025; Scholkmann et al., 2010); (3) reducing the interference of high-frequency noise and low-frequency fluctuation signals using a band pass filter between 0.01 and 0.2 Hz (Wu et al., 2023; Zhou et al., 2024); and (4) converting the filtered optical density data to relative changes of HbO concentration using the modified Beer-Lambert law. To ensure data quality, each channel underwent a signal-to-noise ratio (SNR) check, and channels falling below the threshold or containing severe motion artifacts were excluded. Only channels meeting these quality control criteria were included in subsequent statistical analyses. The preprocessing and quality control procedures were performed independently for each participant under each experimental stimulus condition.

The 14 channels were assigned to four prefrontal regions of interest (ROIs): BA8, BA9, BA10, and BA46. Given that fNIRS primarily reflects hemodynamics in superficial cortical layers, channel assignment was determined by combining device engineering parameters with standardized scalp-to-cortex projection probabilities. For example, CH2 corresponded to the Frontopolar area (BA10) with a probability of 0.8703 and the Orbitofrontal area with a probability of 0.1297, thus primarily attributed to BA10; CH10 corresponded to the Dorsolateral prefrontal cortex (BA9) with a probability of 0.7312, the Frontopolar area with 0.2451, and BA46 with 0.0237, thus significantly attributed to BA9. The above preprocessing steps were repeated for each participant under each experimental condition.

Eye movement data were preprocessed using Data Viewer software, where small fixation points were filtered out and artifacts were further removed. The cleaned data were then exported to Microsoft Excel and analyzed using SPSS. The exported dataset included the following eight key eye movement metrics: Fixation Duration, Fix Pupil, Saccade Amplitude, Saccade Average Velocity, Saccade Duration, Blink Count, Saccade Count, Fixation Count.

### 2.5 Statistical analysis

#### 2.5.1 Eye tracking analysis

To ensure consistency in data analysis, we used the eye movement metrics obtained during the resting-state phase as baseline values and calculated relative changes at each time point accordingly. Positive values indicate higher eye movement activity compared to the resting state, whereas negative values reflect lower activity.

Next, eye movement data from the task phases (excluding gray field intervals) were extracted, and a one-way ANOVA was conducted to test for overall significant differences in eye movement metrics across the three experimental conditions. If the ANOVA results reached statistical significance (*p* < 0.05), an LME was further applied to analyze the temporal trends of the eye movement metrics, thereby revealing the dynamic characteristics of eye movement behavior under different experimental conditions.

#### 2.5.2 fNIRS analysis

The fNIRS data were exported to Microsoft Excel and analyzed in SPSS. To standardize data comparison, the HbO concentration during the resting-state phase was used as the baseline, and relative changes were computed for each ROI. Positive values represent HbO concentrations higher than the baseline, while negative values indicate lower concentrations.

Subsequently, HbO values from the task phases (excluding gray field intervals) were extracted, and a one-way ANOVA was performed to examine differences in both HbO and HbR concentrations across the three experimental tasks within the prefrontal regions. If the ANOVA yielded significant results (*p* < 0.05), an LME was further employed to analyze the temporal evolution of HbO concentration, revealing the activation patterns of the PFC during task execution.

#### 2.5.3 Reaction time analysis

The time interval between consecutive key presses was defined as the reaction time metric, serving as an indicator of participants’ response speed to different experimental stimuli. A one-way ANOVA was then conducted to test for significant differences in reaction time across the three experimental conditions. To provide a more intuitive representation of the distribution characteristics and potential outliers under each condition, corresponding boxplots were generated.

#### 2.5.4 Correlation heatmap analysis

To further explore the relationships between eye movement behavior and brain functional activity, Pearson correlation coefficients were calculated among eye movement metrics, HbO and HbR concentrations, and reaction times. A correlation heatmap was subsequently generated to visually depict the inter-variable correlation patterns, offering insights into the interactions among multimodal indicators and supporting a deeper understanding of their functional associations.

#### 2.5.5 Statistical thresholds and model parameters

All statistical analyses were conducted in SPSS version 25.0 (IBM Corp., Armonk, NY, USA). Unless otherwise stated, tests were two-tailed with a significance level of α = 0.05. For one-way ANOVA, partial η^2^ was reported as the effect size, and significant results were followed by Bonferroni-corrected post-hoc comparisons for three planned contrasts (Color vs. Location, Color vs. Color & Location, and Location vs. Color & Location), with an adjusted significance threshold of α = 0.0167. Pairwise comparisons additionally reported Cohen’s d, and all effect sizes were accompanied by 95% confidence intervals. For linear mixed-effects (LME) models, experimental condition (categorical) and time (continuous) were specified as fixed effects, participant ID was modeled as a random intercept, and the condition × time interaction term was included to examine temporal dynamics between conditions. Models were fitted using maximum likelihood estimation (ML), and residual distributions were assessed for normality via Q–Q plots and Shapiro–Wilk tests.

## 3 Results

### 3.1 Eye tracking

This study systematically examined the effects of three visual stimuli (Color, Location, and Color & Location) on eye movement behavior using a one-way ANOVA followed by Bonferroni-corrected post-hoc tests. As shown in Table 2, significant main effects were observed for Fixation Duration (*F* = 195.688, *p* < 0.001, partial η^2^ = 0.062), Fix Pupil (*F* = 184.458, *p* < 0.001, partial η^2^ = 0.029), Saccade Amplitude (*F* = 408.826, *p* < 0.001, partial η^2^ = 0.129), Saccade Average Velocity (*F* = 325.309, *p* < 0.001, partial η^2^ = 0.021), and Saccade Duration (*F* = 82.784, *p* < 0.001, partial η^2^ = 0.128). However, no significant differences were found in basic eye movement metrics including Blink Count (*p* = 0.518), Saccade Count (*p* = 0.942), and Fixation Count (*p* = 0.944) across the three conditions (*p* > 0.05). The results of the Bonferroni-corrected pairwise comparisons revealed significant differences among all stimuli conditions for Fixation Duration, Fix Pupil, Saccade Amplitude, Saccade Average Velocity, and Saccade Duration (Table 3). Notably, no significant difference was observed between the Location and Color stimuli in terms of Fix Pupil.

**TABLE 2:** One-way ANOVA results of eye movement metrics across three stimuli.

Indicators	*F*	*P*	Indicators	*F*	*P*
Fixation Duration (ms)	195.688	0.000***	Saccade Duration (ms)	82.784	0.000***
Fix Pupil (mm)	184.458	0.000***	Blink Count (counts)	0.661	0.518
Saccade Amplitude (°)	408.826	0.000***	Saccade Count (counts)	0.059	0.942
Saccade Average Velocity (°/s)	325.309	0.000***	Fixation Count (counts)	0.058	0.944

*** denote significance at the 0.1% level.

**TABLE 3:** Comparisons of eye movement metrics between stimuli.

Indicators	Stimult1	Stimult2	Difference	P	95% CI
Fixation Duration	Color	Location	−68.71	0.000***	[−143.15, −31.78]
	Color	Location & Color	−88.968	0.000***	[−109.53, −41.97]
	Location	Location & Color	−20.258	0.000***	[−165.22, −13.72]
Fix Pupil	Color	Location	20.354	1.000	[−106.47, 399.03]
	Color	Location & Color	334.535	0.000***	[25.31, 390.20]
	Location	Location & Color	314.181	0.000***	[47.30, 390.48]
Saccade Amplitude	Color	Location	−0.89	0.000***	[−1.19, −0.32]
	Color	Location & Color	−0.523	0.000***	[−0.92, −0.09]
	Location	Location & Color	0.367	0.000***	[0.09, 0.66]
Saccade Average Velocity	Color	Location	−27.347	0.000***	[−39.50, −6.56]
	Color	Location & Color	−21.346	0.000***	[36.46, −5.71]
	Location	Location & Color	6.001	0.000***	[2.61, 18.62]
Saccade Duration	Color	Location	21.146	0.000***	[7.15, 34.07]
	Color	Location & Color	7.001	0.000***	[2.72, 45.97]
	Location	Location & Color	−14.146	0.000***	[−38.08, −5.92]

*** denote significance at the 0.1% level.

**FIGURE 9:**
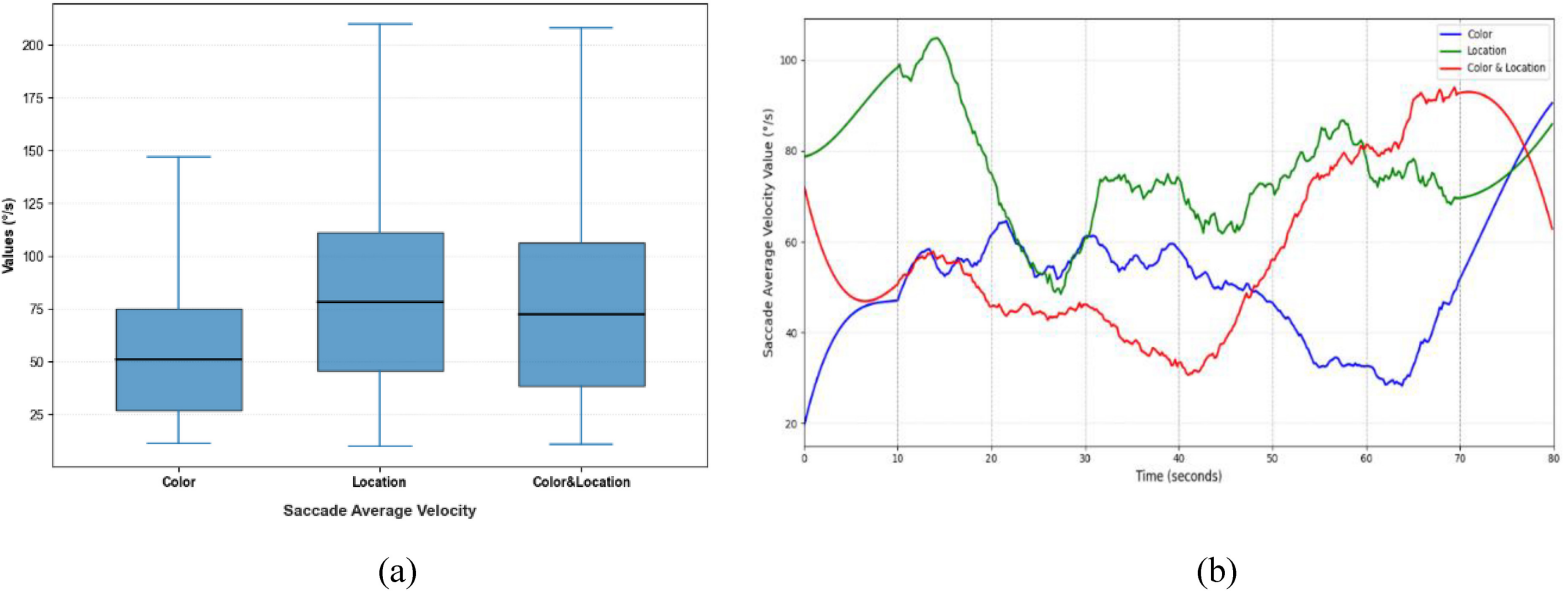
Distribution patterns and temporal trends of saccade average velocity across stimuli. **(a)** Distribution patterns diagram **(b)** temporal trends diagram. Significant differences were observed among the three conditions. Color stimuli showed the lowest median with a narrow range, indicating a conservative scanning strategy; Location stimuli had the highest median with concentrated distribution, reflecting stable eye movements; Color & Location stimuli had an intermediate median but exhibited largest variability in the later phase, indicating inter-individual differences and complex scanning strategies.

**FIGURE 10:**
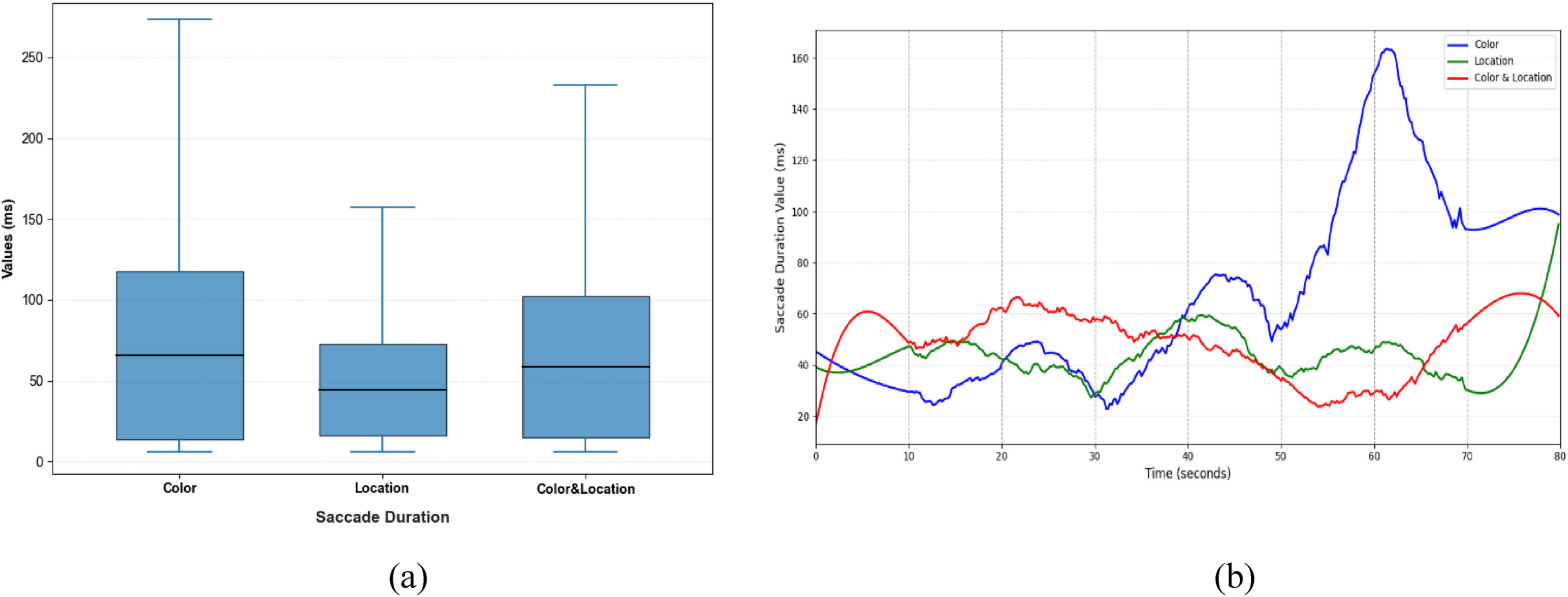
Distribution patterns and temporal trends of saccade duration across stimuli conditions. **(a)** Distribution patterns diagram **(b)** temporal trends diagram. Color stimuli exhibited the longest median and widest range, including multiple extremes; Location stimuli had the shortest median and concentrated distribution; Color & Location stimuli showed a moderate median with a bimodal distribution, reflecting greater individual differences and complex strategies. Temporal trends show increases in later phases for Color stimuli, minor rises for Location stimuli, and fluctuating patterns for Color & Location stimuli.

**FIGURE 11:**
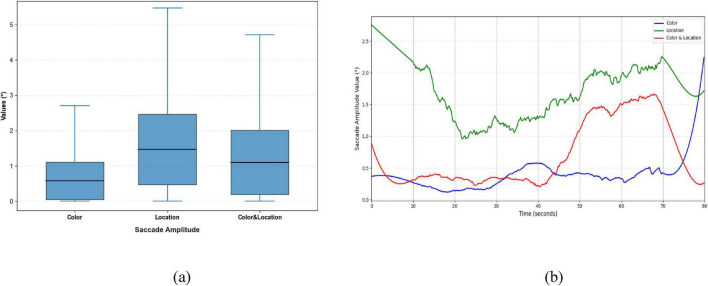
Distribution patterns and temporal trends of saccade amplitude across stimuli conditions. **(a)** Distribution patterns diagram **(b)** temporal trends diagram. Location stimuli had the highest initial amplitude, decreasing to minimum at 20 s, then rising to ∼2.2° in the late phase; Color stimuli remained relatively stable with slight increases at 40 s and 72 s; Color & Location stimuli started at 0.8°, sharply increased after 40 s, and peaked at 1.7° at 68 s, reflecting dynamic differences in spatial gaze range.

To further clarify the regulatory characteristics of each metric under different tasks, the overall distributions and temporal trends of these indicators are described as follows:

Significant differences were observed in Saccade Average Velocity, Saccade Duration, and Saccade Amplitude across the three stimulus conditions (Figures 9–11). Under the Color condition, saccades exhibited significantly lower average velocity and longer duration, reflecting a slower and more variable oculomotor pattern. In contrast, the Location condition elicited the highest velocities with more concentrated distributions, along with more stable fixation durations, suggesting efficient and systematic spatial processing. The Color & Location condition showed the largest fluctuations across velocity, amplitude, and duration, particularly in the later phase of the task, where frequent adjustments indicated increased processing demands and less stable strategies.

**FIGURE 12:**
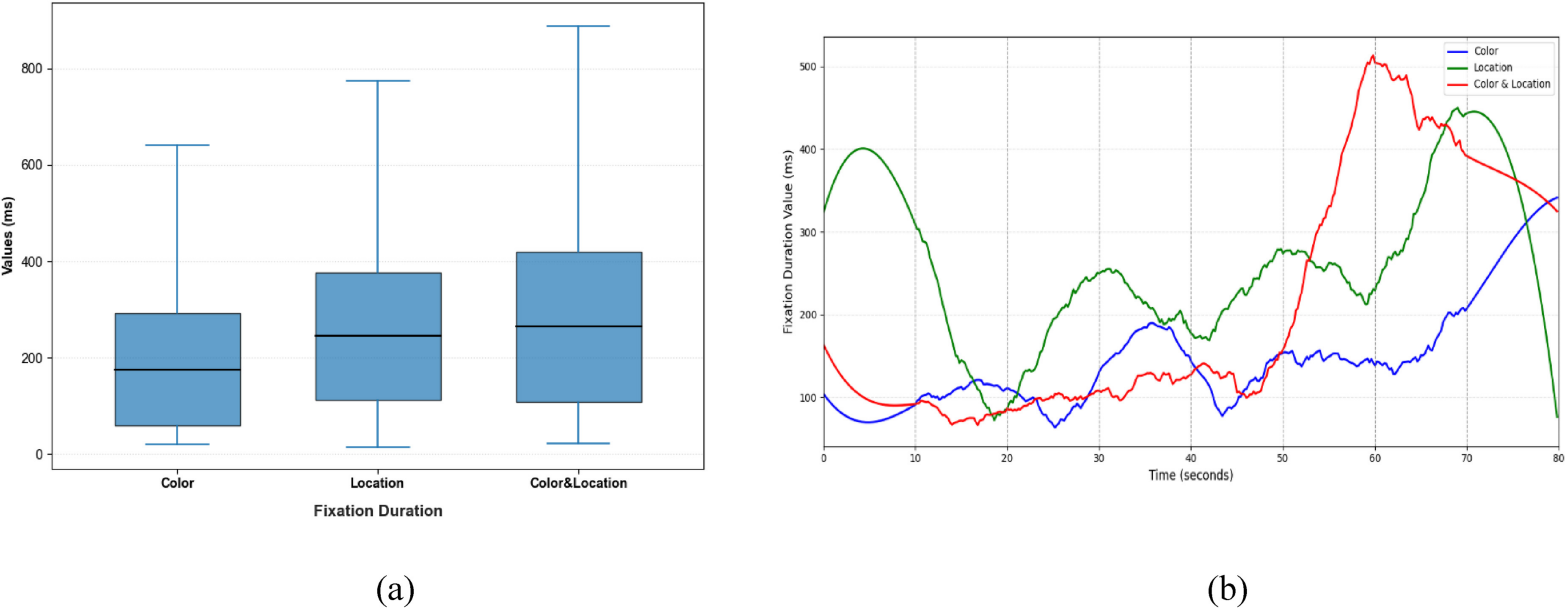
Distribution patterns and temporal trends of fixation duration across stimuli conditions. **(a)** Distribution patterns diagram **(b)** temporal trends diagram. Color stimuli had the shortest median and compact distribution; Location stimuli had a slightly longer median and wider spread; Color & Location stimuli had the longest median with a skewed distribution and maximum exceeding 500 ms, indicating increased cognitive load. Temporal trends show later increases for Color stimuli, minor fluctuations for Location stimuli, and delayed upward trends for Color & Location stimuli.

**FIGURE 13:**
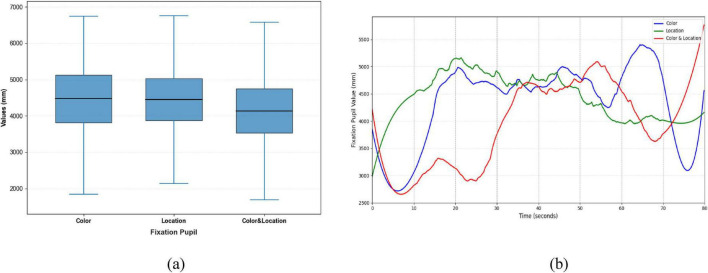
Distribution patterns and temporal trends of fixation pupil across stimuli conditions. **(a)** Distribution patterns diagram **(b)** temporal trends diagram. Color stimuli had the largest median with wide distribution; Location stimuli had a slightly smaller median and concentrated distribution; Color & Location stimuli had the smallest median with high variability, indicating increased cognitive processing load and inter-individual differences. Temporal trends show rapid changes for Color stimuli, Location stimuli, and notable increases in the later phase for Color & Location stimuli.

Clear differences were also evident in Fixation Duration and Pupil responses across the three conditions (Figures 12, 13). The Color condition yielded shorter fixation durations accompanied by significant pupil dilation, suggesting elevated cognitive effort during color-based rivalry. The Location condition showed more stable fixation patterns with less pronounced pupil changes, consistent with reduced cognitive demand. The Color & Location condition produced the longest and most variable fixation durations, together with the largest pupil fluctuations, indicating that combined feature integration imposes the highest attentional load and induces the most complex oculomotor responses.

Taken together, the three types of stimuli produced distinct oculomotor signatures: Color stimuli were associated with slower and more variable saccades coupled with significant pupil dilation; Location stimuli were characterized by the fastest and most stable oculomotor patterns; and Color & Location stimuli elicited the most complex responses, marked by large fluctuations and pronounced individual variability.

### 3.2 fNIRS

Table 4 presents the results of a one-way ANOVA, examining changes in HbO and HbR concentrations in PFC regions during the execution of three different stimuli tasks. The results indicate that HbO and HbR values in the BA8, BA9, and BA10 regions showed statistically significant differences across conditions. In contrast, only HbR values in the BA46 region demonstrated significant statistical differences among the stimuli conditions.

**TABLE 4:** One-way ANOVA results of fNIRS metrics across three stimuli.

ROI (channels)	Label name	Mean-HbO		Mean-HbR	
		***F***	***P***	***F***	***P***
BA8 (CH13, CH14)	FEF	17.186	0.000***	11.627	0.000***
BA9 (CH7, CH8, CH10, CH11, CH12)	DLPFC	19.155	0.000***	25.701	0.000***
BA10 (CH2, CH3, CH4, CH5, CH6, CH9)	FP	7.781	0.000***	42.332	0.000***
BA46 (CH1)	DLPFC	0.703	0.497	14.821	0.000***

*** denote significance at the 0.1% level.

**FIGURE 14:**
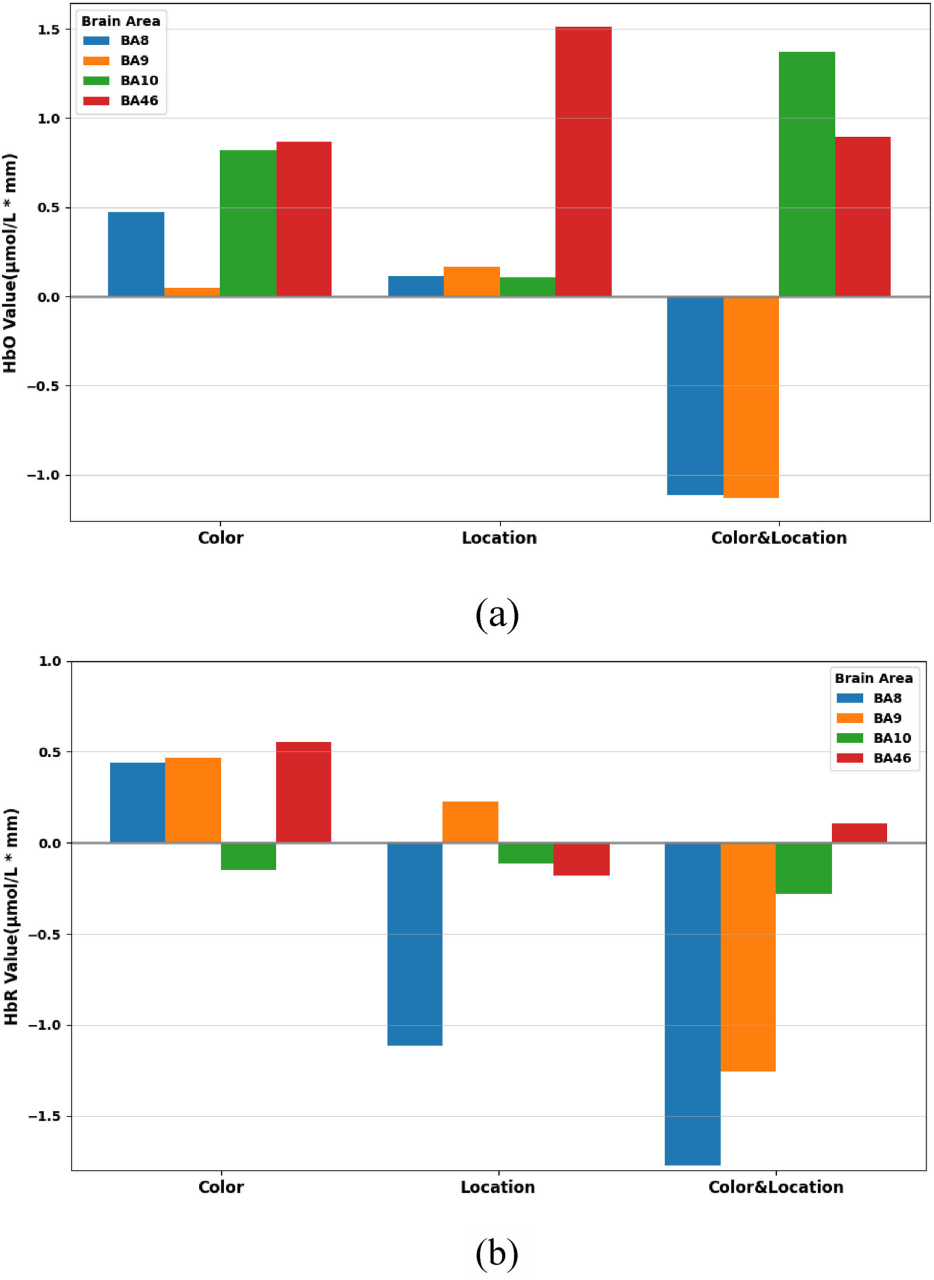
Concentration ranges across brain regions under the three stimuli conditions. **(a)** HbO concentration ranges across BA8, BA9, BA10, and BA46. **(b)** HbR concentration ranges across BA8, BA9, BA10, and BA46.

Through the one-way ANOVA, we further examined the response ranges of prefrontal subregions under different task conditions. Overall results (Figure 14) show that Color stimuli primarily activated BA8 and BA46, characterized by significant changes in both HbO and HbR, as well as parts of BA10; Location stimuli elicited significant responses mainly in BA46; whereas Color & Location stimuli induced the most complex hemodynamic regulation in BA10.

**FIGURE 15:**
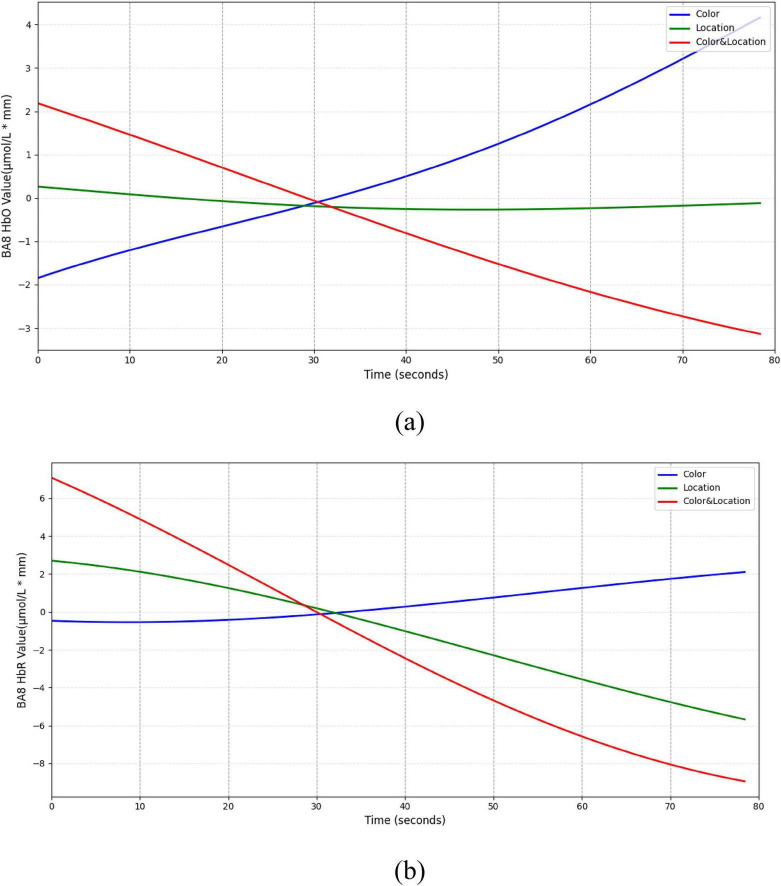
Temporal trend of BA8 under different stimuli conditions. **(a)** HbO concentration temporal trend **(b)** HbR concentration temporal trend. Color stimuli elicited a gradual HbO increase (∼4 μmol/L*mm) with HbR near baseline, Location stimuli showed minimal HbO change and slight HbR decrease (∼–6 μmol/L*mm), and Color & Location stimuli induced concurrent decreases in HbO (∼–30) and HbR (∼–9 μmol/L*mm).

Dynamic trend analysis revealed that each subregion exhibited differentiated hemodynamic patterns over time. In the BA8 region (Figure 15), Color stimuli elicited a sustained increase in HbO accompanied by a slight rise in HbR, indicating prominent attentional modulation under single-feature color processing. In contrast, Location stimuli triggered relatively stable responses, suggesting limited recruitment of BA8 by spatial cues. Under Color & Location stimuli, both HbO and HbR concentrations decreased simultaneously, potentially reflecting attentional resource suppression or depletion during the competitive task.

**FIGURE 16:**
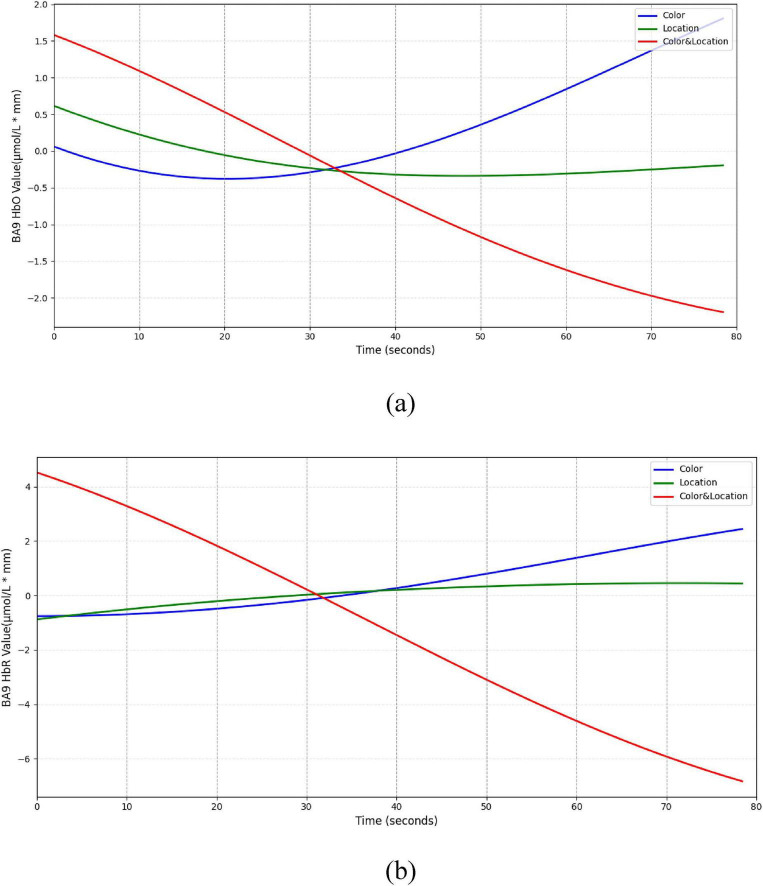
Temporal trend of BA9 under different stimuli conditions. **(a)** HbO concentration temporal trend **(b)** HbR concentration temporal trend. Color stimuli induced an initial slight HbO decrease (∼–0.4 μmol/L*mm) followed by a gradual rise to a peak (∼1.8 μmol/L*mm), with HbR slowly increasing. Location stimuli showed minimal HbO change ( ± 0.5 μmol/L*mm) and slight HbR increase. Color & Location stimuli produced concurrent decreases in HbO (∼–2.5 μmol/L*mm) and HbR (∼–7 μmol/L*mm).

The BA9 region (Figure 16) exhibited a typical “compensatory” pattern. Under Color stimuli, HbO initially decreased slightly but gradually increased, suggesting additional resource mobilization for processing conflicting information. Location stimuli produced minimal changes, maintaining low-level activity. In the Color & Location condition, HbO and HbR concentrations continuously declined, indicating possible deactivation under high-load integrative processing.

**FIGURE 17:**
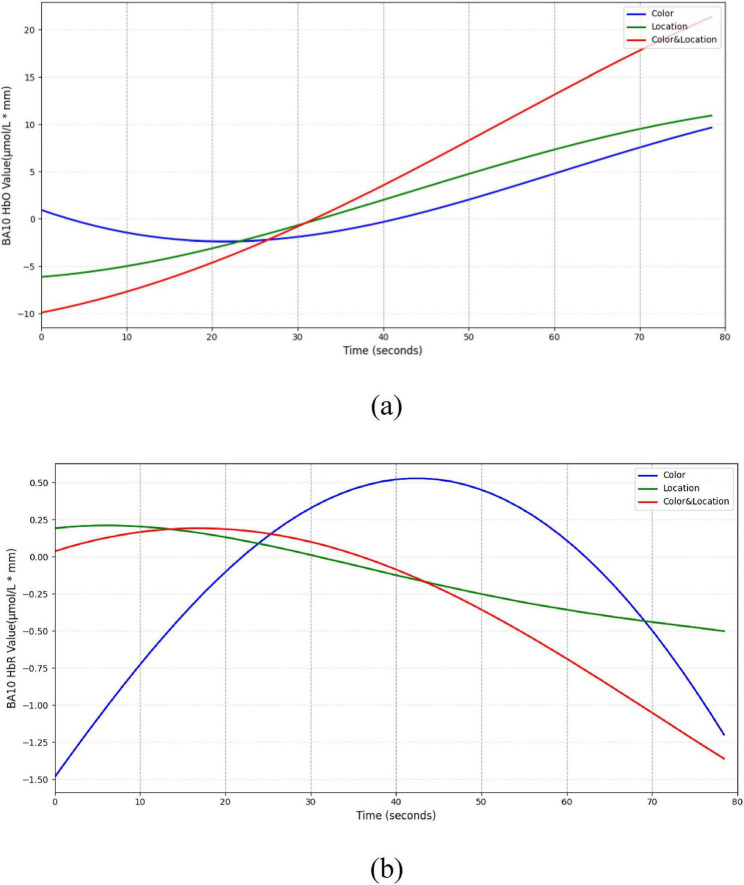
Temporal trend of BA10 under different stimuli conditions. **(a)** HbO concentration temporal trend **(b)** HbR concentration temporal trend. Color stimuli induced a gradual HbO increase, reaching 10 μmol/L*mm, with slight HbR decrease. Location stimuli produced a moderate HbO rise (∼11 μmol/L*mm) and slow HbR decline (∼–0.5 μmol/L*mm). Color & Location stimuli elicited the strongest response, with rapid HbO increase (∼22 μmol/L*mm) and marked HbR decrease (< –1 μmol/L*mm).

In the BA10 region (Figure 17), both Color and Location stimuli induced increases in HbO, whereas the Color & Location condition produced the most pronounced response, with peak HbO significantly higher than other conditions and HbR markedly decreased. These results suggest that BA10 plays a central role in multisource information integration and higher-order cognitive control.

**FIGURE 18:**
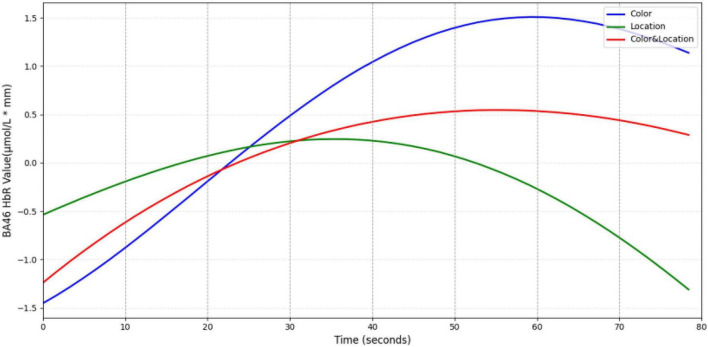
Temporal trend of BA46 under different stimuli conditions. Color stimuli elicited the fastest HbR increase, peaking at ∼1.5 μmol/L mm around 60 s before declining. Location stimuli showed the slowest rise, reaching a peak of ∼0.25 μmol/L⋅mm at 35 s, followed by a rapid decrease. Color & Location stimuli induced a moderate peak (∼0.5 μmol/L⋅mm), which gradually returned to baseline. These results indicate that HbR accumulation in BA46 exhibits distinct stimulus-dependent temporal characteristics.

The BA46 region (Figure 18) exhibited biphasic dynamics in HbR concentration. Under Color stimuli, HbR rose slowly but reached the highest peak; Location stimuli elicited rapid increase followed by quick decline; Color & Location stimuli showed intermediate amplitude. This biphasic pattern may reflect stimulus-specific differences in the initiation and inhibition rhythms during executive control.

In summary, prefrontal subregions exhibited both intensity and time-dependent functional differentiation under different visual stimuli. BA8 showed strong responses to Color stimuli but remained stable under Location stimuli. BA9 displayed compensatory effects under Color stimuli. BA10 exhibited the strongest HbO activation under Color & Location stimuli; and BA46 demonstrated biphasic dynamic responses. These findings reveal distinct regulatory mechanisms of Color, Location, and their interaction on prefrontal subregions during binocular rivalry tasks.

### 3.3 Reaction time

**FIGURE 19:**
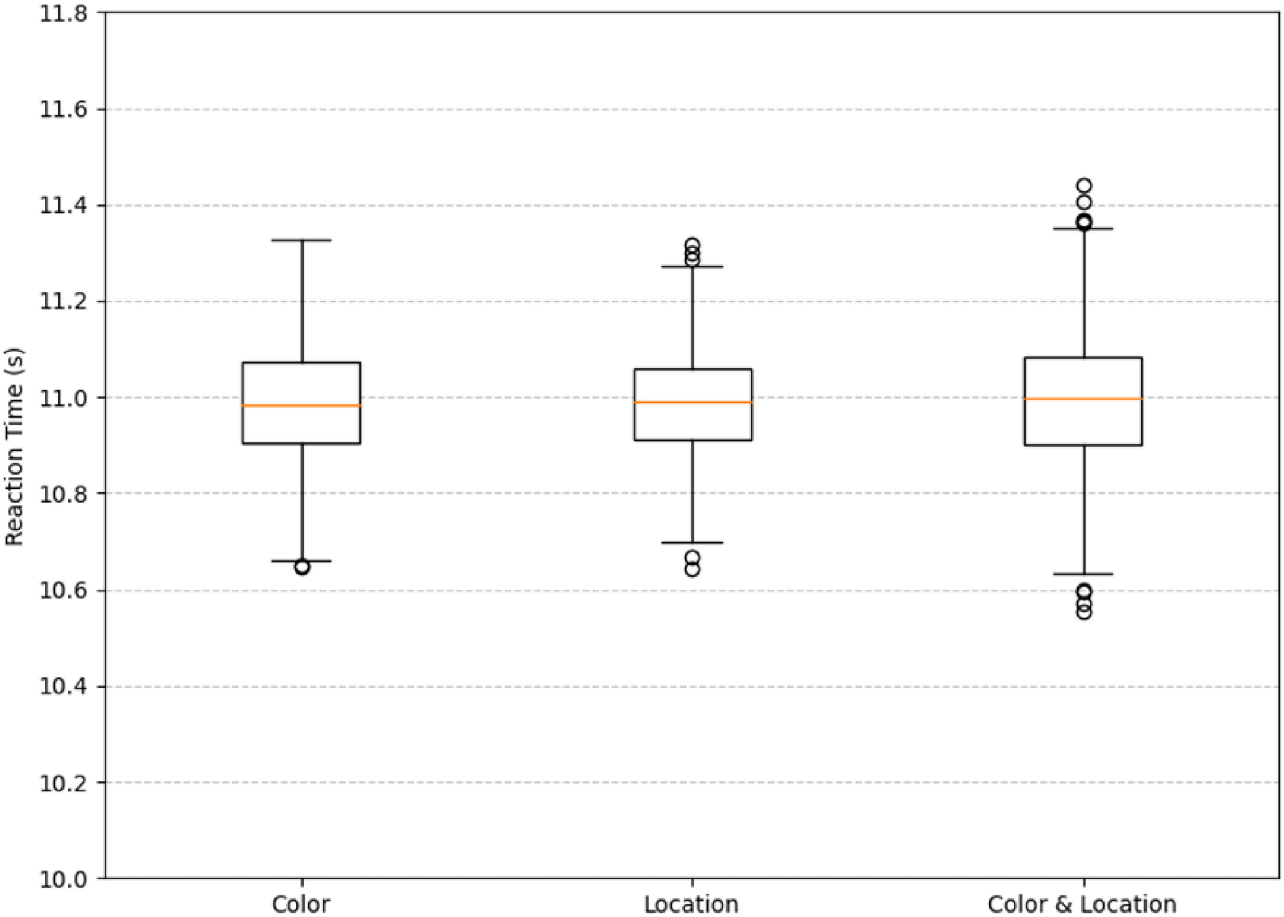
Boxplot of reaction time under difference conditions. The median reaction times across all three conditions are close to 11.0 s, and the overall distributions are relatively concentrated.

The boxplot illustrates the distribution of reaction times under three different stimuli conditions. As shown in the Figure 19, the median reaction times across all three conditions are close to 11.0 s, and the overall distributions are relatively concentrated.

Statistical analysis using a one-way ANOVA revealed no significant differences in reaction time among the Color, Location, and Color & Location conditions, F (2, 2197) = 0.001, *p* = 0.999, partial η^2^ < 0.001. Bonferroni-corrected pairwise comparisons confirmed the absence of significant differences between any two conditions (all *p* = 1.000; 95% CI ranges all included 0). Although no statistically significant differences were observed, we further examined the descriptive statistics to explore potential trends in response variability across conditions.

Specifically, under the Color stimuli, the interquartile range is slightly wider with fewer outliers. Under the Location stimuli, the interquartile range is the narrowest, but more outliers are observed. Under the Color & Location stimuli, the interquartile range is moderately wide, and the number of outliers is the highest, indicating a greater variability in reaction times. This may be attributed to the increased task complexity, which leads to larger individual differences in processing composite information. In contrast, under the single-stimuli conditions, reaction times are more consistent, suggesting lower task difficulty and more similar response speeds among participants. Although the median reaction times are comparable across all three conditions, the number of outliers and the spread of the interquartile ranges indicate that the Color & Location stimuli impose a higher cognitive load. This condition likely requires more attentional resources and cognitive processing, resulting in significant variations in individual response times.

### 3.4 Correlation heatmap

**FIGURE 20:**
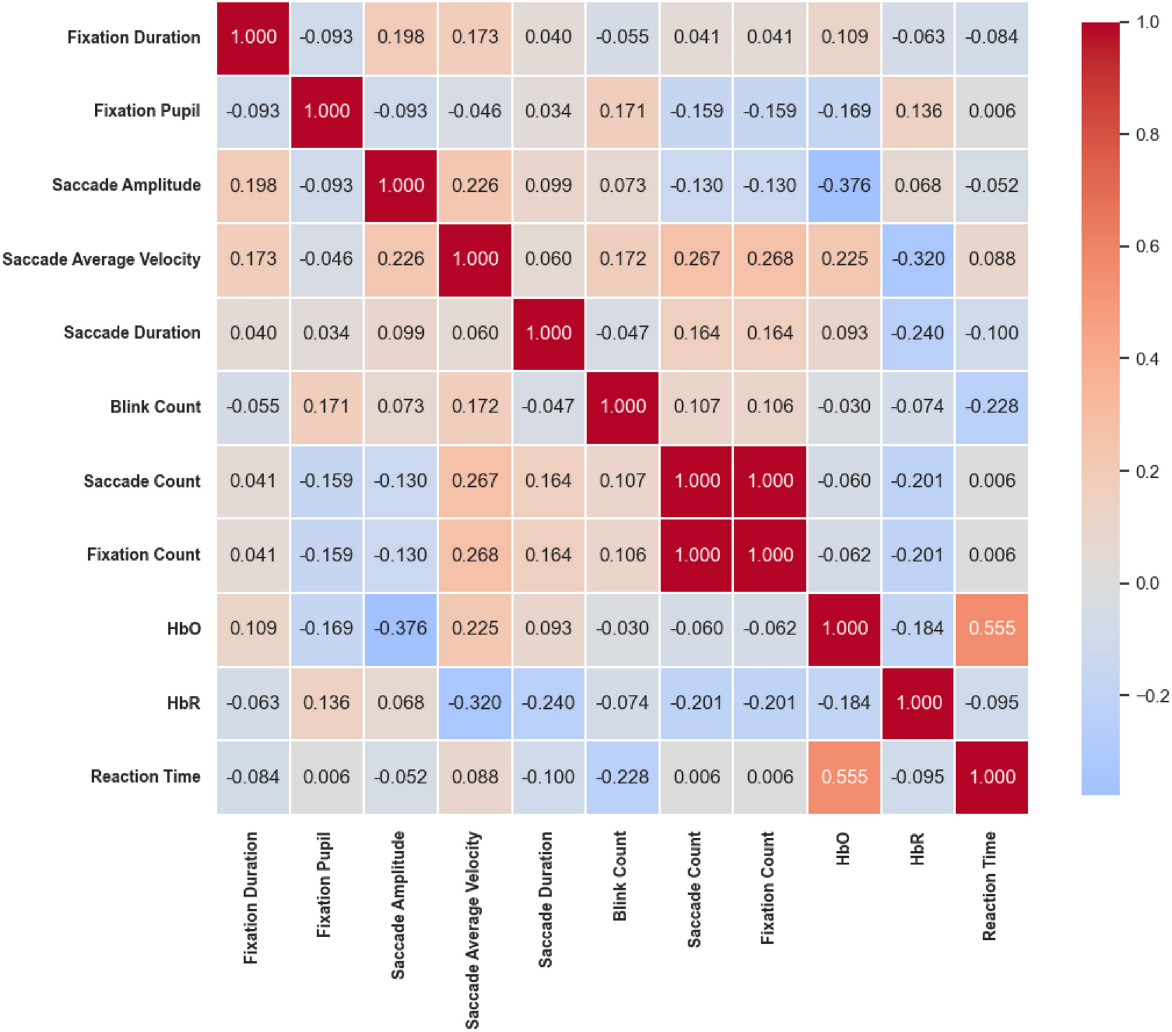
Correlation heatmap of reaction time under the Color stimuli. Overall correlations among eye movement metrics were weak, but moderate-to-strong coupling was observed between eye movement and cerebral hemodynamics. HbO was positively correlated with reaction time (*r* = 0.555, *p* < 0.01) and negatively with saccade amplitude (*r* = –0.376, *p* < 0.01); HbR was negatively correlated with saccade average Velocity (*r* = –0.320, *p* < 0.05).

Under the Color stimuli (Figure 20), overall correlations among eye movement indicators were weak; however, moderate-to-strong coupling was observed between eye movement and cerebral hemodynamic signals. Specifically, HbO was significantly positively correlated with Reaction Time (*r* = 0.555, *p* < 0.01) and significantly negatively correlated with Saccade Amplitude (*r* = –0.376, *p* < 0.01). In addition, HbR was significantly negatively correlated with Saccade Average Velocity (*r* = –0.320, *p* < 0.05). These findings suggest a close interaction between hemodynamic responses and oculomotor behavior under color competition.

**FIGURE 21:**
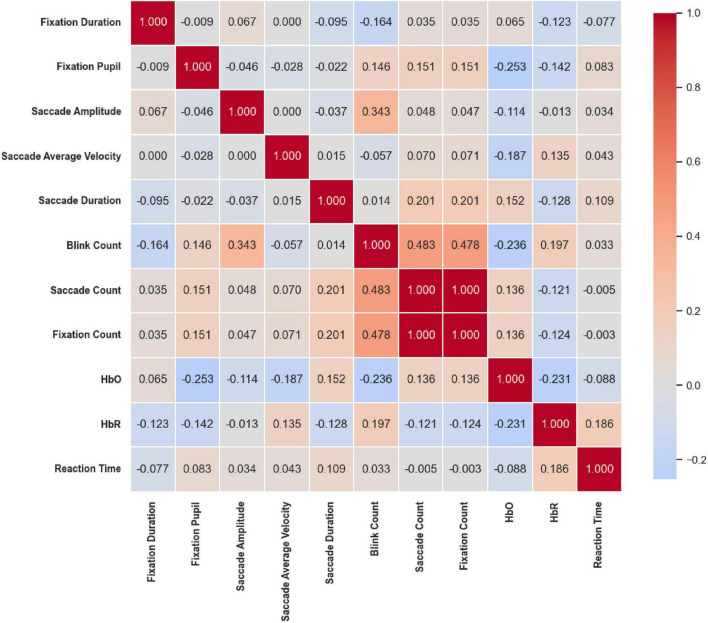
Correlation heatmap of reaction time under the Location stimuli. Moderate-to-strong correlations were mainly among eye movement frequency measures: Saccade count and fixation count were perfectly correlated (*r* = 1.000, *p* < 0.01) and both moderately correlated with Blink Count (*r* = 0.483 and 0.478, *p* < 0.01). Prefrontal hemodynamic indicators showed weak associations with eye movements.

Under the Location stimuli (Figure 21), moderate-to-strong correlations were primarily concentrated among eye movement frequency measures. Saccade Amplitude was moderately positively correlated with Blink Count (*r* = 0.343, *p* < 0.01), while Saccade Count and Fixation Count were perfectly correlated (*r* = 1.000, *p* < 0.01). Both were also moderately positively correlated with Blink Count (*r* = 0.483 and *r* = 0.478, respectively; *p* < 0.01). In contrast, prefrontal hemodynamic indicators showed only weak correlations with eye movement parameters, suggesting that spatial cues mainly influence oculomotor frequency coupling, with limited involvement of hemodynamic regulation.

**FIGURE 22:**
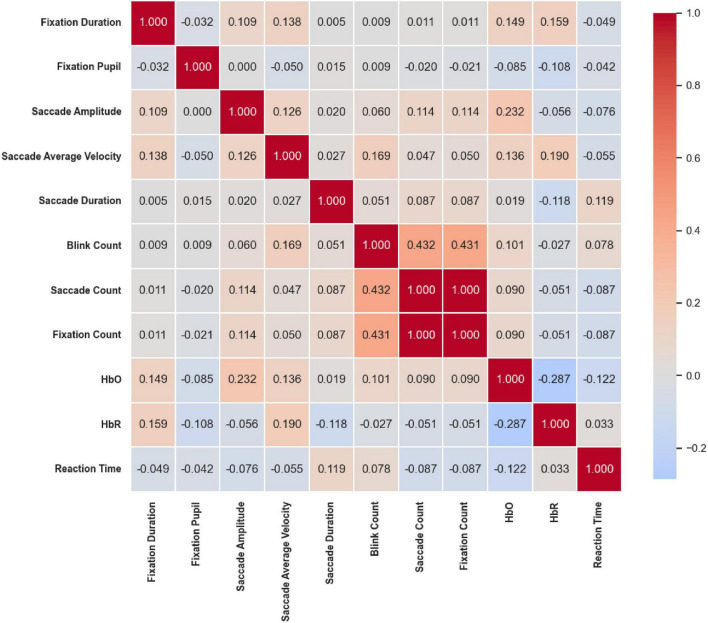
Correlation heatmap of reaction time under Color & Location stimuli. Frequency coupling resembled the Location condition, with saccade count and fixation count perfectly correlated (*r* = 1.000, *p* < 0.05) and both positively correlated with blink count (*r* ≈ 0.43, *p* < 0.05). Correlations between eye movements and hemodynamics were generally weak or non-significant.

Under the Color & Location stimuli (Figure 22), a similar pattern of frequency coupling was observed. Blink Count was significantly positively correlated with both Saccade Count (*r* = 0.432, *p* < 0.05) and Fixation Count (*r* = 0.431, *p* < 0.05), and Saccade Count and Fixation Count were perfectly correlated (*r* = 1.000, *p* < 0.05), consistent with the results observed under the Location condition. Beyond these frequency-based associations, correlations between eye movement and hemodynamic indicators were generally weak or non-significant.

In summary, across all three stimuli conditions, strong internal coupling among eye movement indicators was consistently observed. Saccade Amplitude was positively correlated with Saccade Velocity, while Saccade Count and Fixation Count were nearly perfectly synchronized. Conversely, Fixation Pupil size tended to be negatively correlated with both Saccade Amplitude and Velocity. Regarding cerebral hemodynamics, under the Color condition, increased Saccade Amplitude was associated with decreased HbO levels and prolonged Reaction Time. Under both the Location and Color & Location conditions, faster saccadic dynamics and higher blink frequency were negatively correlated with HbO, while the antagonistic relationship between HbO and HbR remained robust across all tasks. Overall, these results highlight a consistent coupling pattern between oculomotor dynamics, pupillary responses, and prefrontal hemodynamic activity, with variations in strength and direction depending on task dimensions.

## 4 Discussion

By integrating eye-tracking and fNIRS data, this study systematically examined prefrontal functional differentiation under Color, Location, and Color & Location stimuli. The study aimed to test the following hypotheses: (1) Stimuli involving color changes will elicit saccadic responses and increased prefrontal cognitive load, particularly reflected in significant activation of the VLPFC;

(2) Stimuli involving location changes will preferentially activate the dorsal pathway to support efficient spatial information updating and maintenance;(3) When both color and location cues change simultaneously, the visual system will face intensified resource rivalry and processing bottlenecks, manifested as prolonged reaction times co-occurring with widespread hyperactivation in the PFC.

Multimodal evidence under the Color stimuli supported Hypothesis 1. Participants exhibited “low-velocity, high-stability” Saccades Velocity (median ∼50°/s, low variability), consistent with the theory that color feature extraction requires active modulation of visual system wavelength sensitivity (Witzel and Gegenfurtner, 2018). pupil dilation was significantly positively correlated with task demand, indicating increased cognitive resource allocation. fNIRS data revealed significant increases in HbO in prefrontal regions including FEF and DLPFC, supporting the notion of heightened prefrontal cognitive load. Reaction times were strongly positively correlated with HbO (*r* = 0.62, *p* < 0.01), reflecting that the fine integration of color features, such as hue and saturation, is both time-consuming and highly dependent on prefrontal resources (Liu et al., 2025).

Results under the Location stimuli supported Hypothesis 2. Saccade Velocity was the fastest (median ∼78°/s) and highly consistent, while Saccade Amplitude remained stable (∼1.2° ± 0.3°) and Fixation Durations were prolonged but low in variability (∼230 ms), consistent with Posner’s (1980) automatic spatial attention network model. These findings suggest that Location stimuli processing relies on low-load, automated dorsal pathway mechanisms (Corbetta and Shulman, 2002; Verga and Kotz, 2019). fNIRS data showed minimal HbO fluctuation in dorsal pathway regions (e.g., intraparietal sulcus, superior frontal gyrus; 0.0000–0.0002 mmol/L⋅mm), and overall prefrontal activation was lower than that observed in the Color stimuli, consistent with the neural efficiency hypothesis that high-efficiency processing is accompanied by reduced neural activity (Neubauer and Fink, 2009).

Multimodal evidence under the Color & Location stimuli supported Hypothesis 3. Saccade Velocity (median ∼72°/s), Amplitude (initial suppression 0–50 s, sudden increase after 50 s), and Duration (bimodal distribution) exhibited pronounced fluctuations, which intensified over time (Saccade Velocity peaked after 60 s), consistent with dual-task bottleneck theory (Pashler, 1994). Reaction time distributions were the broadest and increased in the later stages (∼700 ms), in line with the notion that dual-feature rivalry impairs performance and induces prefrontal overactivation (Ding et al., 2024). fNIRS revealed widespread prefrontal HbO increases (e.g., BA10, BA46; peak 0.02 mmol/L mm) and biphasic HbR dynamics (initial increase followed by decrease), indicating resource competition-induced overactivation and reduced processing efficiency.

Overall, results suggest a “task complexity-driven functional differentiation” pattern:

(1) Simple tasks: Color and Location stimuli involve single-feature processing. Color stimuli require prefrontal engagement (FEF/DLPFC) for fine feature integration, whereas Location stimuli rely primarily on automated dorsal pathway processing (Corbetta and Shulman, 2002). Both show relatively stable behavior, but prefrontal activation patterns differ—Color stimuli exhibit “high activation–moderate stability,” while Location stimuli show “low activation–high stability.” (2) Complex tasks: Color & Location stimuli involve multi-feature integration. Prefrontal regions participate in higher-level integration (DLPFC conflict resolution) and resource allocation (FEF saccade strategy adjustments), resulting in increased activation and reduced behavioral stability (higher Reaction time variability).

This pattern aligns with Petrides (2005) prefrontal functional subdivision model, in which subregions display task-specific activation as cognitive demand increases. However, direct evidence for BA10 as a “core hub” was not observed (e.g., no significant functional connectivity enhancement), and further studies using TMS or high spatiotemporal resolution imaging (e.g., iEEG-fNIRS) are warranted.

In conclusion, the data reveal that task complexity (from single-feature to combined-feature integration) is associated with prefrontal activation patterns: Color stimuli increase prefrontal cognitive load for feature integration, Location stimuli rely on dorsal pathway efficiency, and Color & Location stimuli induce widespread prefrontal activation and processing bottlenecks. These findings provide empirical support for attentional resource allocation mechanisms in integrating visual features and spatial information. Study limitations include: (1) this study did not incorporate analyses of fixation stability and micro saccades; future research could combine high-resolution eye tracking to further examine their contributions; (2) interpretations of “rapid saccades” were primarily based on mean values and trends, lacking direct support from latency distributions; subsequent studies could adopt distributional analyses to strengthen the robustness of the conclusions; (3) the experiment did not segment the task into early, middle, and late phases, leaving the operational definition of neural efficiency insufficiently precise; (4) the causal roles of distinct prefrontal subregions remain unclear and require validation using interventional techniques (e.g., TMS); and (5) under the combined Color & Location stimuli, individuals may employ different attentional strategies, and such variability warrants further investigation at the individual level.

## 5 Conclusion

This study systematically revealed the division of labor and coordination mechanisms between visual attention and PFC activation under Color, Location, and combined Color & Location visual stimuli by integrating eye-tracking, fNIRS, and reaction time data. The results indicate that BA10 plays a core hub role in multi-feature integration, while BA46 is primarily involved in the continuous updating and regulation of spatial information. The dynamic coupling between eye movement behavior and PFC hemodynamic responses reveals the flexible attentional resource allocation strategies employed by the prefrontal cortex under complex task conditions.

Specifically, Color stimuli induced rapid saccadic behavior and relatively higher prefrontal load, Location stimuli promoted efficient spatial encoding via the dorsal pathway, while the Color & Location combined stimulus increased cognitive load, as evidenced by greater dispersion in reaction times and enhanced cortical activation. These results provide empirical support for the differentiation of visual attention and prefrontal functions, although the specific role of BA46 requires further validation. Future studies will incorporate neuroimaging techniques with higher spatial resolution to explore this in greater depth.

In summary, this research not only deepens our understanding of visual attention mechanisms and functional differentiation within the prefrontal cortex but also offers a solid theoretical foundation and physiological markers—such as BA10 and BA46 activation patterns, saccade dynamics, and hemodynamic responses—for multimodal cognitive state monitoring and adaptive optimization of BCI systems.

Future studies could further incorporate machine learning techniques to enhance the predictive accuracy of integrated eye-tracking and fNIRS data. Additionally, extending these findings to clinical populations and tasks involving multisensory conflicts may help validate their practical applicability and broaden their use in both cognitive neuroscience and adaptive human-machine system design.

## Data Availability

The datasets presented in this study can be found in online repositories. The names of the repository/repositories and accession number(s) can be found below: https://doi.org/10.6084/m9.figshare.29551169.v1.
